# Molecular recognition and packing frustration in a helical protein

**DOI:** 10.1371/journal.pcbi.1005909

**Published:** 2017-12-19

**Authors:** Loan Huynh, Chris Neale, Régis Pomès, Hue Sun Chan

**Affiliations:** 1 Department of Biochemistry, University of Toronto, Toronto, Ontario, Canada; 2 Department of Physics, Applied Physics, and Astronomy, Rensselaer Polytechnic Institute, Troy, New York, United States of America; 3 Molecular Medicine, The Hospital for Sick Children, Toronto, Ontario, Canada; 4 Department of Molecular Genetics, University of Toronto, Toronto, Ontario, Canada; University of Chicago, UNITED STATES

## Abstract

Biomolecular recognition entails attractive forces for the functional native states and discrimination against potential nonnative interactions that favor alternate stable configurations. The challenge posed by the competition of nonnative stabilization against native-centric forces is conceptualized as frustration. Experiment indicates that frustration is often minimal in evolved biological systems although nonnative possibilities are intuitively abundant. Much of the physical basis of minimal frustration in protein folding thus remains to be elucidated. Here we make progress by studying the colicin immunity protein Im9. To assess the energetic favorability of nonnative versus native interactions, we compute free energies of association of various combinations of the four helices in Im9 (referred to as H1, H2, H3, and H4) by extensive explicit-water molecular dynamics simulations (total simulated time > 300 μs), focusing primarily on the pairs with the largest native contact surfaces, H1-H2 and H1-H4. Frustration is detected in H1-H2 packing in that a nonnative packing orientation is significantly stabilized relative to native, whereas such a prominent nonnative effect is not observed for H1-H4 packing. However, in contrast to the favored nonnative H1-H2 packing in isolation, the native H1-H2 packing orientation is stabilized by H3 and loop residues surrounding H4. Taken together, these results showcase the contextual nature of molecular recognition, and suggest further that nonnative effects in H1-H2 packing may be largely avoided by the experimentally inferred Im9 folding transition state with native packing most developed at the H1-H4 rather than the H1-H2 interface.

## Author summary

Biomolecules need to recognize one another with high specificity: promoting “native” functional intermolecular binding events while avoiding detrimental “nonnative” bound configurations; i.e., “frustration”—the tendency for nonnative interactions—has to be minimized. Folding of globular proteins entails a similar discrimination. To gain physical insight, we computed the binding affinities of helical structures of the protein Im9 in various native or nonnative configurations by atomic simulations, discovering that partial packing of the Im9 core is frustrated. This frustration is overcome when the entire core of the protein is assembled, consistent with experiment indicating no significant kinetic trapping in Im9 folding. Our systematic analysis thus reveals a subtle, contextual aspect of biomolecular recognition and provides a general approach to characterize folding frustration.

## Introduction

Molecular recognition is the basis of biological function. For different parts of the same molecule or different molecules to recognize one another, a target set of interactions need to be favored while other potential interactions are disfavored. Biomolecules accomplish these simultaneous tasks via the heterogeneous interactions encoded by their sequences. For proteins, such energetic heterogeneity is enabled but also constrained by a finite alphabet of twenty amino acids. Thus the degree to which non-target interactions can be avoided through evolutionary optimization is limited [1, 2]. Conflicting favorable interactions, referred to as frustration, are often present in biological systems. From a physical standpoint, it is almost certain that some of the frustration is a manifestation of the fundamental molecular constraint on adaptation, although under certain circumstances frustration can be exploited to serve biological function [3, 4].

Protein folding entails intra-molecular recognition. Early simulations suggested that nonnative contacts can be common during folding [5]. This predicted behavior applies particularly to models embodying a simple notion of hydrophobicity as the main driving force [6, 7]. Experimentally, however, protein folding is thermodynamically cooperative [7, 8]. Folding of many single-domain proteins does not encounter much frustration from nonnative interactions in the form of kinetic traps [9]. Celebrated by the consistency principle [10] and the principle of minimal frustration [11], these empirical trends have inspired Gō-like modeling, wherein native-centric interactions are used in lieu of a physics-based transferable potential [12–14]. Extensions of this approach allow nonnative interactions to be treated as perturbations in a largely native-centric framework [15–17]. The success of these models poses a fundamental challenge to our physical understanding as to why, rather non-intuitively, natural proteins are so apt at avoiding nonnative interactions. Solvation effects must be an important part of the answer [18], as has been evident from the fact that coarse-grained protein models incorporating rudimentary desolvation barriers exhibit less frustration and higher folding cooperativity than models lacking desolvation barriers [7, 19, 20]. More recently, and most notably, folding of several small proteins has been achieved in molecular dynamics studies with explicit water [21, 22]. Nonnative contacts are not significantly populated within sections of the simulated trajectories identified as folding transition paths [23] though they do impede conformational diffusion [24]. These advances suggest that certain important aspects of protein physics are captured by current atomic force fields, although they still need to be improved to reproduce the high degrees of folding cooperativity observed experimentally [22, 25–28].

In this context, it is instructive to ascertain how atomic force fields, as they stand, disfavor nonnative interactions, so as to help decipher molecular recognition mechanisms in real proteins. We take a step toward this goal by comparing the stabilities of native and nonnative configurations of fully formed helices from a natural protein. By construction, this approach covers only a fraction of all possible nonnative configurations and therefore only provides, albeit not unimportantly, a lower bound on the full extent of frustration. Nonetheless, because of its focus on tractable systems, we obtain a wealth of reliable simulation data from which physical insights are gleaned. We do so by applying explicit-water molecular dynamics simulations to compute potentials of mean force (PMFs) between various helices [29] of the *E*. *coli* colicin immunity protein Im9 [30]. Im9 is a small single-domain protein that undergoes two-state-like folding [31, 32] to a native structure with four helices packed around a hydrophobic core [33]. Its folding mechanism and that of its homolog Im7 have been extensively characterized experimentally [30–40] and theoretically [41–46]. Of particular relevance to our study are experimental Φ-value analyses suggesting that the Im9 folding transition state has a partially formed hydrophobic core stabilized by interactions between helix 1 (H1) and helix 4 (H4), whereas helix 3 (H3) adopts its native conformation only after the rate-limiting step of folding [32]. These experimental inferences have since been rationalized by simulations showing that H1 and H4 are formed whereas about one half of helix 2 (H2) remains unstructured in the Im9 transition state [41], and that, unlike Im7, there is no significant kinetic trap along the Im9 folding pathway [45, 46].

Building on these advances, our systematic PMF analysis provides a hitherto unknown perspective on these hallmarks of Im9 folding. Notably, we found significant packing frustration between H1 and H2, viz., a nonnative packing orientation can achieve a lower free energy than that afforded by the native packing of these two helices in isolation. Superficially, this simulation result seems at odds with experiments indicating little frustration in Im9 folding. On closer examination, however, our discovery provides an unexpected rationalization for experiments indicating that folding is initiated by the more stabilizing H1-H4 interactions rather than by H1-H2 packing. Because the H1-H2 packing frustration can be circumvented by following such a kinetic order, our finding suggests that the Im9 folding pathway might have evolved to avoid a potential H1-H2 kinetic trap. This example underscores that the inner workings of molecular recognition can be rather subtle and deserves further exploration, as will be elaborated below.

## Results

**Fig 1:**
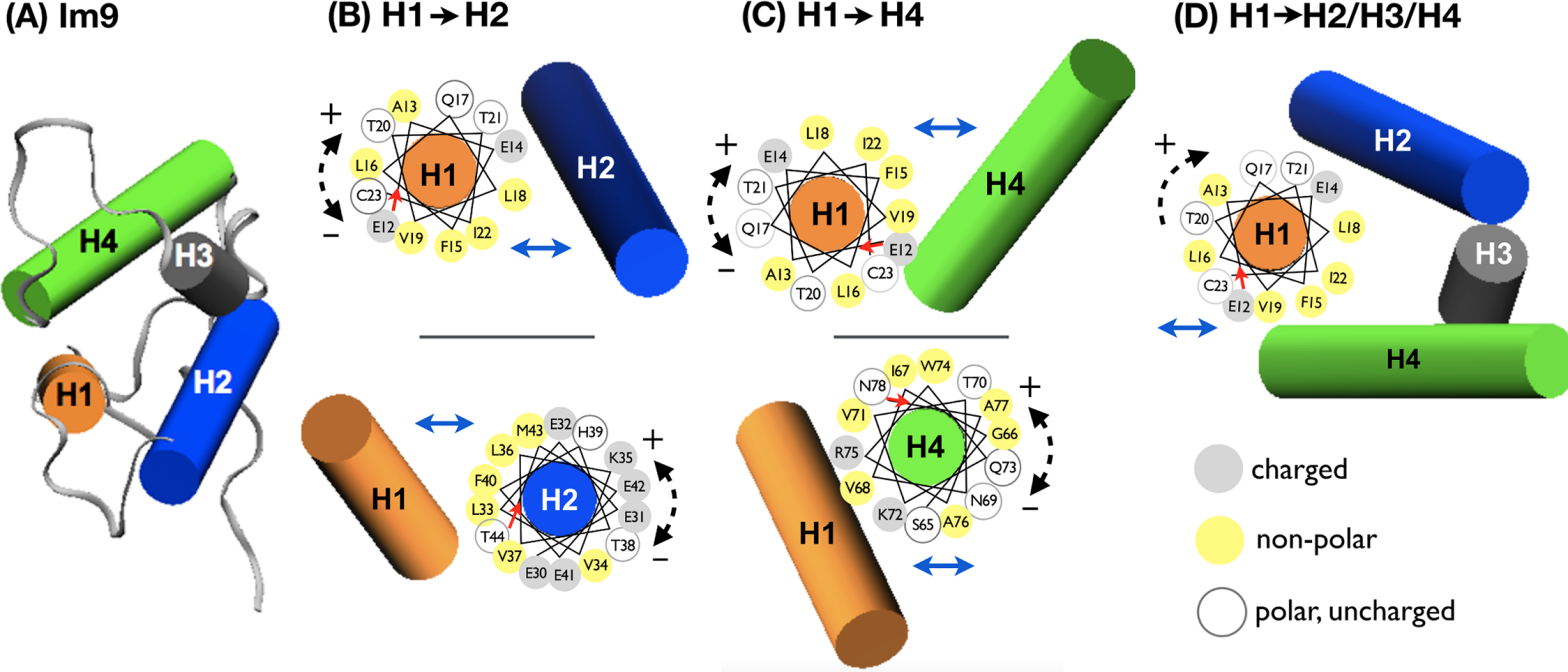
Schematics of Im9 simulation systems. (A) Full-length Im9 (PDB ID: 1IMQ [47]). Helices are represented as cylinders. (B-D) Combined helical wheel and cylinder representations of systems wherein H1 packs against (B) H2, (C) H4, or (D) H2, H3, and H4. For each helical wheel, the red arrow indicates the residue closest to the viewer. Energetic effects of translating H1 in the directions of the solid blue arrows are determined with the position(s) and orientation(s) of the opposing helix or helices (cylinders) fixed. To evaluate the energetic consequences of helical rotation and nonnative packing, the fragment depicted by the helical wheel is rotated (dashed arrows) to nonnative orientations with positive (+) and negative (‒) rotation angles. Residues on the helical wheels are colored differently depending on the type of amino acid: charged residues in grey, nonpolar residues in yellow, and polar residues in white.

With the above rationale in mind, we apply the technique described in *Methods* and **S1 Text** for extensive molecular dynamics simulations to study the 86-residue helical protein Im9 [47], focusing primarily on the interactions among various sets of fragment(s) comprising one or more helices. For terminological simplicity, each fragment set in an interacting pair—including a single helix—is referred to as a bundle below. PMFs of nine pairs of bundles (Fig 1 and Table 1) are computed to ascertain whether native or nonnative associations are preferred. Although intra-bundle conformational variations are restricted in most of our model systems (*Methods*), the studied configurations are all physically realizable. It follows logically that the observation of favorable nonnative packing in our simulations is sufficient to demonstrate, at least for the atomic force field used here, that favorable nonnative interactions do exist in Im9.

**Table 1:** Im9 simulation systems.

**System Identifier**^**a**^	**Im9 Residues**^**b**^
H1→H2	12–23, 30–44
H1→H4	12–23, 65–78
H1→^N^H4	12–23, 62–78^c^
H1→H2/H4	12–23, 30–44, 65–78
H1→H2/^N^H4	12–23, 30–44, 62–78^c^
H1→H2/H4^C^	12–23, 30–44, 65–86
H1→H2/^N^H4^C^	12–23, 30–44, 62–86^c^
H1→H2/H3/H4	12–23, 30–44, 50–55, 65–78
H1→H2^L^H3^L^H4^C^	12–23, 30–86
H1^L^H2	12–44^d^

^a^The two interacting bundles in each system are separated by an arrow. Superscripts “N” and “C” represent, respectively, the three residues N-terminal to H4 and the eight residues C-terminal to H4. Superscript “L” represents the loop residues connecting two consecutive helices (e.g., in H2^L^H3^L^H4^C^, the first “L” stands for residues 24–29, and the second “L” stands for residues 45–49) whereas a slash between two helix-containing fragments (blocks of residues) indicates that the chain segment between the fragments is not part of the bundle.

^b^Helical residue selection is based on DSSP [48], except H3, which is extended from 51–54 to 50–55 based on Friel *et al*.[32].

^c^Note that Asp62, Ser63, and Pro64 are part of this extended H4 fragment.

^d^At variance with the other systems, the H1^L^H2 system allows free reorientation of the helical interface.

### Helices 1 and 2 favor nonnative packing in isolation

We begin by investigating the free energy landscape for the association of H1 with H2, a packing interaction that accounts for the largest two-helix interface in the native state of Im9, burying 5.3 nm^2^ or 17% of the total surface area of H1 and H2. Throughout this study, surface areas of helical bundles are computed as the solvent-accessible surface areas of the given bundles in isolation, irrespective of the solvent exposure of the configurations in the complete Im9 folded structure. Using an enhanced sampling technique known as umbrella sampling with virtual replica exchange (US-VREX, see *Methods*) for restrained helical configurations at systematically varied target packing angles, we compute PMFs for H1-H2 association in the absence of their intervening loop (the H1→H2 system in Fig 1B and Table 1). The PMFs are determined for the native orientation as well as for nonnative orientations and nonnative crossing angles entailed by the imposed rotational preferences (*Methods* and *S1 Text*). Our technique allows these simulations to converge rapidly (**S1 Fig**). Each PMF is then integrated over a free-energy basin to provide a binding free energy, Δ*G*_bind_, for a specific inter-helix geometry.

**Fig 2:**
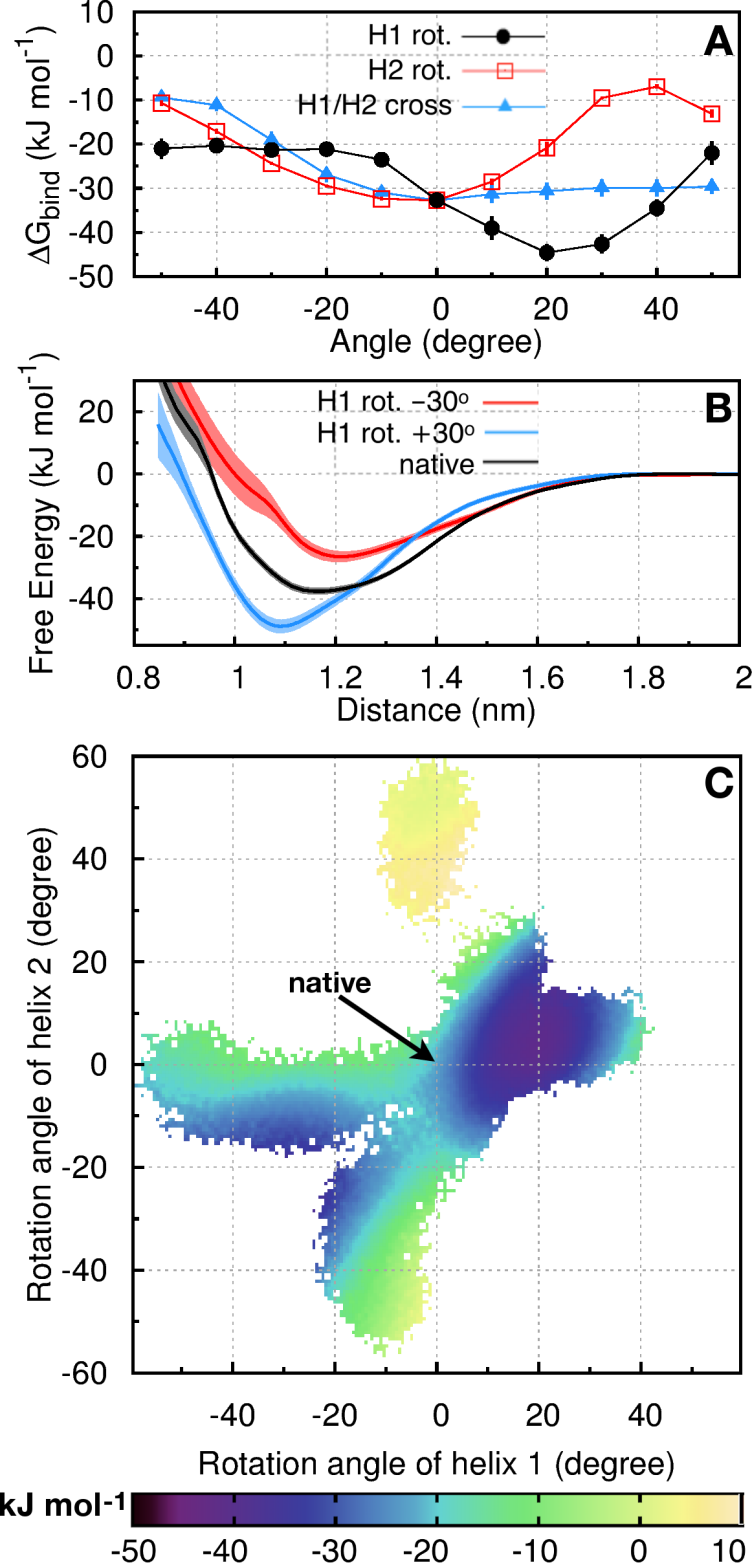
Im9 binding free energies for H1→H2. (A) Binding free energies, Δ*G*_bind_, for the association of H1 and H2 with native and nonnative packing angles. Nonnative configurations are generated by rotating H1 (filled black circles), or H2 (open red squares), or changing the H1-H2 crossing angle (filled blue triangles). Δ*G*_bind_ is computed from the total Boltzmann-weighted H1-H2-distance-dependent population of the entire free-energy basin (thus it correlates with but is not necessarily equal to the minimum PMF value; see **S1 Text**). (B) PMFs here are distance-dependent free energies for the association of H1 and H2 in native (black curve) and nonnative orientations with H1 targeted to be rotated by +30° (blue curve) or −30° (red curve). Actual rotation angles sampled during the computations of these PMFs are close to the targets (**S3 Fig**). Standard deviations of the mean from block averaging are shown as vertical bars in (A) or shaded regions in (B). (C) Two-dimensional PMF of the H1, H2 packing angles in simulations with helical rotation but no change in H1-H2 crossing angle. Data are drawn from multiple simulations, including one started and restrained to the native orientation and twenty others with preferred nonnative packing angles in which one helix is rotated by ±10–50°. Each free energy value (bottom color scale) plotted is the minimum of the distance-dependent PMF for a given inter-helix geometry (**S1 Text**). White regions have no sampling. By construction, the H1-H2 distance at the minimum of PMF can be different for different rotation angles (see example in **S4 Fig**). It is noteworthy that the two free-energy basins exhibited here are nonetheless robustly observed at essentially the same packing angles in multiple restrained simulations wherein inter-helical distances are targeted at a given $d_{i}^{0}$ ranging from 1.0 nm to 1.3 nm (**S5 Fig**).

Unexpectedly, H1-H2 association is favored by a 20–30° positive rotation of H1 against H2. Binding in this nonnative orientation is 10–12 kJ/mol more stable than that in the native orientation (black circles in Fig 2A and Table 2), a free energy difference equivalent to a ~50-fold increase in bound population (**S1 Text**). In contrast, the binding free energy profiles for rotating H2 against H1 (Fig 2A, red squares) or changing the H1-H2 crossing angle (Fig 2A, blue triangles) indicate that the state corresponding to native packing (0° angle in Fig 2A) is situated well within the basin of lowest free energy with respect to these degrees of freedom, although a ≤50° positive change in H1-H2 crossing or a ≤20° negative rotation of H2 against H1 would leave the system approximately iso-energetic with the native packing (Fig 2A). As mentioned, these binding energies are computed from PMFs such as those in Fig 2B and **S2 Fig**.

**Table 2:** Binding free energies for Im9 systems in native and nonnative orientations.

		Δ*G*_bind_ (kJ/mol)		
System^a^	Rotated helix	Native	+30° Rotation	ΔΔ*G*_bind_ (kJ/mol)^b^
H1→H2	H1	‒33 ± 0.3	‒43 ± 1	‒10 ± 1
H1→H2	H2	‒33 ± 0.3	‒10 ± 0.1	23 ± 0.3
H1→H4	H1	‒28 ± 1	‒22 ± 1	6 ± 1
H1→H4	H4	‒28 ± 1	‒33 ± 1	‒5 ± 1
H1→^N^H4	H1	‒44 ± 1	‒22 ± 1	22 ± 1
H1→^N^H4	^N^H4	‒44 ± 1	‒21 ± 2	23 ± 2
H1→H2/H4	H1	‒49 ± 1	‒71 ± 0.1	‒22 ± 1
H1→H2/^N^H4	H1	‒80 ± 5	‒78 ± 3	2 ± 6
H1→H2/H4^C^	H1	‒55 ± 2	‒54 ± 2	1 ± 3
H1→H2/^N^H4^C^	H1	‒67 ± 4	‒53 ± 5	14 ± 6
H1→H2/H3/H4	H1	‒68 ± 3	‒79 ± 1	‒11 ± 3
H1→H2^L^H3^L^H4^C^	H1	‒75 ± 1	‒62 ± 3	13 ± 3

^a^Each row represents a pair of interacting bundles. One bundle is the reference. The other, i.e., those along the “Rotated helix” column, is rotated. The relative positions of all C_α_ atoms within any given bundle—which include C_α_ atoms in the loop and/or terminal regions if they are part of the bundle—are maintained at the corresponding relative positions in the PDB structure of the entire protein. The absolute position of the reference bundle is fixed in a global Cartesian coordinate system by harmonic position restraints on all of its C_α_ atoms along all three—**x**, **y**, and **z**—axes of the coordinate system. For the rotated bundle, the position restraints are applied only along the **y** and **z** axes. This serves to fix the relative angular orientation of the two bundles but allow for a variable distance between them. Center-of-mass distance between the reference and rotated bundles is varied during simulations by changing the favored **x**-value of the one-dimensional harmonic restraint on the rotated bundle. See *Methods*.

^b^ΔΔ*G*_bind_ = Δ*G*_bind_(+30° rotation) ‒ Δ*G*_bind_(native); negative values of ΔΔ*G*_bind_ indicate that +30° rotation is more favorable than the native orientation.

A broader view of the orientation-dependent H1-H2 packing free energy landscape can be seen in Fig 2C. Instead of fixing either H1 or H2 in its native orientation (as in Fig 2A), Fig 2C provides the relative favorability of packing orientations resulting from simultaneous rotations of H1 and H2. This two-dimensional PMF is generated by combining sampling data for H1 and H2 rotations under harmonic biasing potentials (**S1 Text**). It is clear from this two-dimensional landscape that native packing [(H1, H2) rotations equal (0°, 0°)] is less favored than the free energy minimum at (+19°, +4°). Indeed, this minimum is situated in a rather broad basin encompassing many nonnative orientations with *simultaneous* H1 rotation from approximately +5° to +25° and H2 rotation from approximately ‒3° to +15° that are energetically more favorable than the native H1-H2 orientation (0°, 0°). Fig 2C reveals further that there exists another basin of favorable nonnative H1-H2 packing for which both helices rotate by approximately ‒20°. In short, our systematic analysis in Fig 2 demonstrates unequivocally that packing frustration exists in Im9, in that when H1 and H2 are considered in isolation, nonnative packing is favored over native packing.

To assess the prospect that intervening loop residues may provide additional guidance for native packing of H1 against H2, we also simulate this helix-loop-helix as a single chain (H1^L^H2 system; Table 1). Because the covalent connection of H1 to H2 is incompatible with the large helical separations used in our importance sampling, we study the H1^L^H2 system without inter-helical distance bias in simulations initiated in either the native state or one of 20 different nonnative orientations in which H1 or H2 is rotated by ±10–50°. [Because the actual rotations sampled during simulations are close to those targeted by the restraining potentials (**S4 Fig**), we do not distinguish between target and actual rotations hereafter]. Although these simulations do not converge to a single conformational distribution, they show broad sampling of H1 rotation with a stable or metastable state near +20° rotation of H1, even when simulation is initiated at the native packing angle (**S6 Fig**).

### But helix 1 is favored to pack natively against the rest of the protein

To explore how the H1-H2 packing frustration might be overcome in Im9 folding, we next investigate the impact of the rest of the protein on the packing between H1 and H2 by computing binding free energies for the association of H1 and H2 not in isolation but in the presence of additional protein fragments involving the other two helices H3 and H4 as well as loop and terminal residues. The conformations of the loop and terminal residues in our simulations are restrained to those in the Im9 PDB structure.

We first consider the association of H1 with a bundle comprising helices 2, 3, and 4 connected by their intervening loops and extending to the protein's C-terminus (H1→H2^L^H3^L^H4^C^; Table 1). Interestingly, for this system, native packing is found to be 13 ± 3 kJ/mol more favorable than the nonnative packing resulting from a +30° rotation of H1 (Table 2). The very fact that a nonnative rotation of H1 is substantially favored in H1→H2 (Fig 2A and Table 2) but disfavored in H1→H2^L^H3^L^H4^C^ (Table 2) demonstrates clearly that some components of the H2^L^H3^L^H4^C^ bundle besides H2 are crucial for overcoming the H1-H2 packing frustration and guiding H1 to pack natively. Furthermore, because native packing is favored in H1→H2^L^H3^L^H4^C^ despite the residues N-terminal to H1 (including a short 3–10 helix) being excluded in this model system, these N-terminal residues are likely not necessary for ensuring native packing of H1 against the rest of the Im9 protein.

### H3 and loop residues surrounding H4 assist native packing of H1 in varying degrees

We now dissect the H2^L^H3^L^H4^C^ bundle to ascertain the contributions from different parts of this bundle to native H1 packing. To this end, binding free energies for the association of H1 with a variety of subsets of H2^L^H3^L^H4^C^ are computed. We first consider a bundle comprising helices 2 and 4 (H1→H2/H4; Table 1). Somewhat surprisingly, native packing in the H1→H2/H4 system is disfavored by as much as 22 ± 1 kJ/mol when compared against nonnative packing with H1 rotated by +30°, even more than the corresponding nonnative preference of 10 ± 1 kJ/mol for H1→H2 (Table 2). This observation implies that H4 by itself is not promoting H1-H2 native packing and therefore H3, loops, and/or the C-terminus must be responsible for driving native packing of H1 with H2^L^H3^L^H4^C^. Indeed, when compared against H2/H4, the presence of these other elements in H2^L^H3^L^H4^C^ results in a 26 ± 1 kJ/mol preference for native H1 packing and a 9 ± 3 kJ/mol discrimination against nonnative H1 packing with a +30° rotation (Table 3).

**Table 3:** Differences between H1 binding free energies for different Im9 helical bundles in native and nonnative orientations.

H1-interacting Fragment		ΔΔ*G*_bind_ (kJ/mol)^a^	
A	B	Native	+30° H1 Rotation
H2	H2/H4	‒16 ± 1	‒28 ± 1
H4	H2/H4	‒21 ± 1	‒49 ± 1
^N^H4	H2/^N^H4	‒36 ± 5	‒56 ± 3
H2/H4	H2/^N^H4	‒31 ± 5	‒7 ± 3
H2/H4	H2/H4^C^	‒6 ± 2	17 ± 2
H2/H4	H2/^N^H4^C^	‒18 ± 4	18 ± 5
H2/H4	H2/H3/H4	‒19 ± 3	‒8 ± 1
H2/H4	H2^L^H3^L^H4^C^	‒26 ± 1	9 ± 3

^a^ΔΔ*G*_bind_ = Δ*G*_bind_(H1,fragment B) ‒ Δ*G*_bind_(H1,fragment A); negative values of ΔΔ*G*_bind_ indicate that fragment B packs more favorably against H1 than does fragment A in the noted orientation.

To better pinpoint the role of H3 in this intra-molecular recognition process, we compute binding free energies for the association of H1 and a bundle comprising helices 2, 3 and 4 but without the intervening loops and the C-terminus (H1→H2/H3/H4; Fig 1D and Table 1). For this model system, native packing is less favorable than +30° rotation of H1 by 11 ± 3 kJ/mol (Table 2). Nonetheless, in comparison to H1→H2/H4, the inclusion of H3 favors native packing more than it favors nonnative packing with a +30° rotation of H1 (Table 2). This observation indicates that H3 is capable of correcting part of the nonnative tendencies of H1 imparted by its interactions with a bundle comprising only of H2 and H4; but H3 is insufficient to ensure native packing in the absence of the connecting loops and/or the C-terminus.

To explore whether inclusion of residues neighboring H4 may alter its effect on H1-H2 packing, we consider three residues immediately N-terminal to H4 (Asp62, Ser63, and Pro64). These residues are chosen because they are known to associate directly with H1 in the NMR structure [47] and thus they may contribute positively to native intra-molecular recognition. Consistent with this expectation, once these three residues are included, the H1-binding free energies in the resulting H1→H2/^N^H4 system (Table 1) for native packing and nonnative +30° rotation of H1 become essentially energetically equivalent (ΔΔ*G*_bind_ = 2 ± 6 kJ/mol; Table 2). Inasmuch as promoting native H1-binding is concerned, this represents a significant improvement over H1→H2/H4 that favors the +30°-rotated nonnative packing by 22 ± 1 kJ/mol (Table 2). Indeed, in the context of H1→H2/H4, addition of these N-terminal flanking residues assists native packing by 31 ± 5 kJ/mol, much more than the 7 ± 3 kJ/mol increase in stability they also impart on the nonnative packing of H1 with a +30° rotation (Table 3). These numbers underscore the important role of Asp62, Ser63, and Pro64 in discriminating against nonnative packing of H1.

Another set of helix-flanking residues that may assist native packing in Im9 is its C-terminus. Such an effect is expected because a +30° rotation of H1 would likely place its constituent residue Phe15 into a steric clash with the C-terminal residue Phe83 (**S7 Fig**) and thus existence of the C-terminus should discriminate against such a rotation of H1. To evaluate this hypothesis, we compute H1-binding free energies with a bundle comprising H2 and H4 as well as the protein's C-terminus (H1→H2/H4^C^; Table 1). Similar to the addition of Asp62, Ser63, and Pro64 N-terminal to H4 in H2/^N^H4 bundle, inclusion of the C-terminus in H2/H4^C^ eliminates the strong nonnative bias in H1→H2/H4, resulting in essentially no discrimination between the native orientation and a +30° rotation of H1 (ΔΔ*G*_bind_ = 1 ± 3 kJ/mol; Table 2). Relative to H1→H2/H4, addition of the C-terminus not only favors native packing by 6 ± 2 kJ/mol but also directly disfavors +30° rotation of H1 by 17 ± 2 kJ/mol (Table 3). The latter penalization of nonnative packing (which does not occur in H1→H2/^N^H4) is consistent with the aforementioned steric consideration (**S7 Fig**).

Interestingly, the native-promoting effects of N- and C-terminal extensions to H4 are essentially additive. When both extensions are added to H4, the H2/^N^H4^C^ system (Table 1) is sufficient to favor native packing of H1 by 14 ± 6 kJ/mol over the nonnative packing with +30° rotation of H1 (Table 2).

### Native packing between H1 and H4 is assisted by flanking loop residues

**Fig 3:**
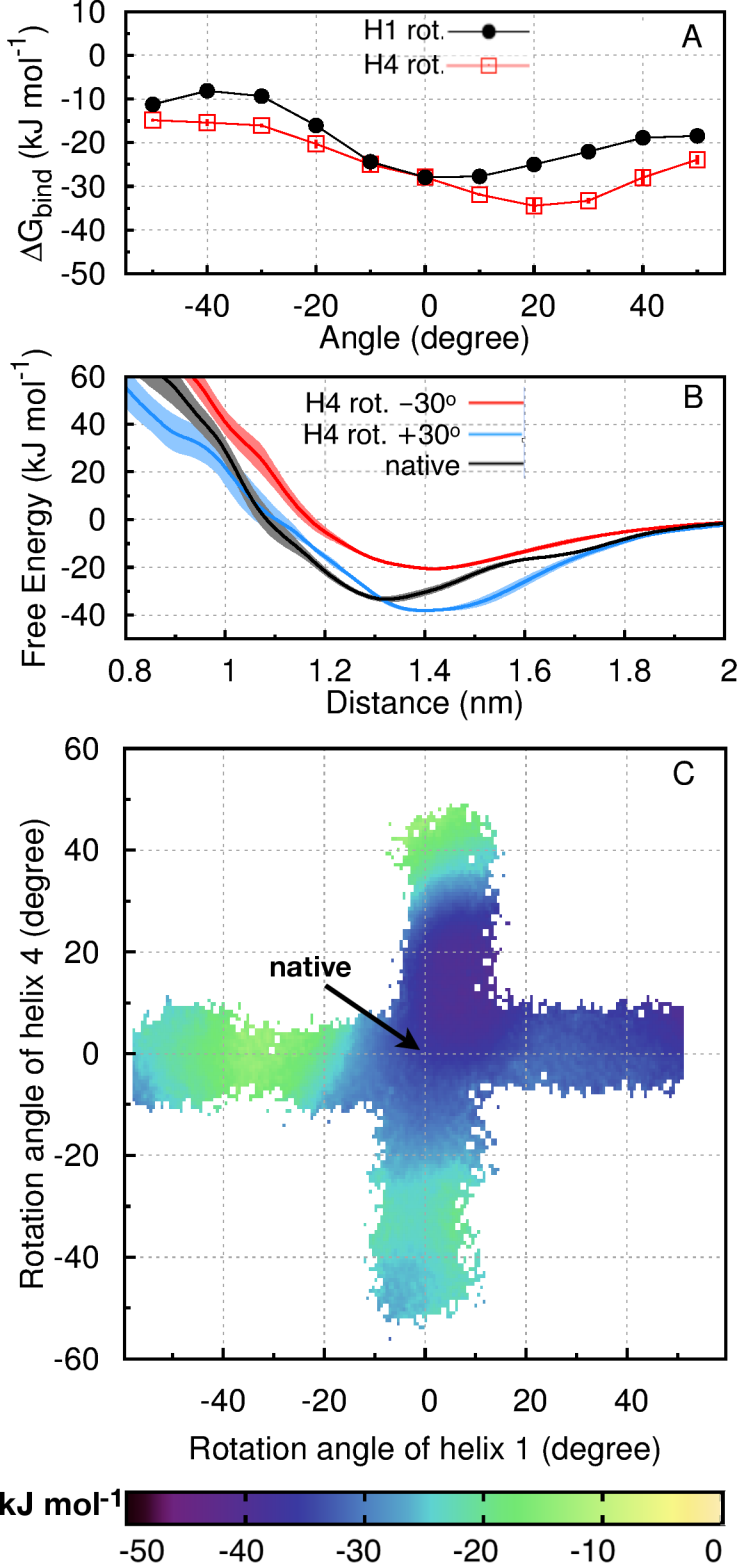
Im9 binding free energies for H1→H4. (A) Binding free energies, Δ*G*_bind_, for the association of H1 and H4 with native and nonnative packing angles generated by rotating H1 (filled black circles), or H4 (open red squares). Δ*G*_bind_ is computed from multiple PMF values as in Fig 2 (**S1 Text**). (B) PMFs describing distance-dependent free energies for the association of H1 and H4 in native (black curve) and nonnative orientations of H4 rotated by +30° (blue curve) or −30° (red curve). Standard deviations of the mean from block averaging are shown as vertical bars (A) or shaded regions (B). (C) Two-dimensional PMF of H1 and H4 packing angles, constructed by the same procedure as that in Fig 2C (**S1 Text**). White regions have no sampling.

After analyzing systems involving H2, we now turn to the intra-molecular recognition between H1 and H4 without involving H2. Native H1-H4 packing constitutes the second largest two-helix interface in the Im9 folded structure, burying 3.7 nm^2^ which amounts to 13% of the sum of individual surface areas of H1 and H4. PMFs for helices 1 and 4 in isolation (H1→H4; Fig 1C and Table 1) are computed in the native orientation as well as nonnative orientations resulting from rotations of H1 or H4. When H1 is rotated while H4 is fixed, native packing is favored (Fig 3A, black circles); however, when H4 is rotated with H1 fixed, a +30° nonnative rotation of H4 leads to 5 ± 1 kJ/mol stabilization (decrease in Δ*G*_bind_) relative to native (red squares in Fig 3A and Table 2). Distance-dependent PMFs for the native orientation and ±30° rotations of H4 are shown in Fig 3B, indicating that the favored nonnative packing at +30° is attained at an H1-H4 separation slightly larger than native by about 0.1 nm. The two-dimensional PMF (Fig 3C) as a function of H1 and H4 rotation angles shows further that native H1-H4 packing (0°, 0°) is situated at the periphery of a broad basin of favored orientations centered roughly around (+10°, +10°). The same two-dimensional landscape suggests that H1 rotations of ≥ +50° or ≤ ‒50° can also be favored with little or no H4 rotation.

We noted earlier that a 3-residue N-terminal extension to H4 directly contacts H1 in the native state and that the inclusion of these residues assisted the native packing of H1 against a bundle comprising helices H2 and H4. Consistent with that observation, these three residues—Asp62, Ser63, and Pro64—likewise assist the native packing of H1 against H4, viz., their inclusion in the H1→^N^H4 system (Table 1) makes native packing (Δ*G*_bind_ = ‒44 ± 1 kJ/mol) significantly more favorable than the nonnative packing with a +30° rotation of H4 (Δ*G*_bind_ = ‒21 ± 2 kJ/mol) while still favoring native orientation of H1 (Table 2). We conclude from these results that helices H1 and H4 are nearly capable of associating in native-like conformations by themselves in isolation; and that they can certainly achieve native packing with the assistance from the 3-residue N-terminal extension to H4. These results suggest that Im9 residues 12–23 and 62–78 may serve as major components of a native-like folding nucleus.

### Certain specific interactions are particularly favorable to nonnative packing

**Fig 4:**
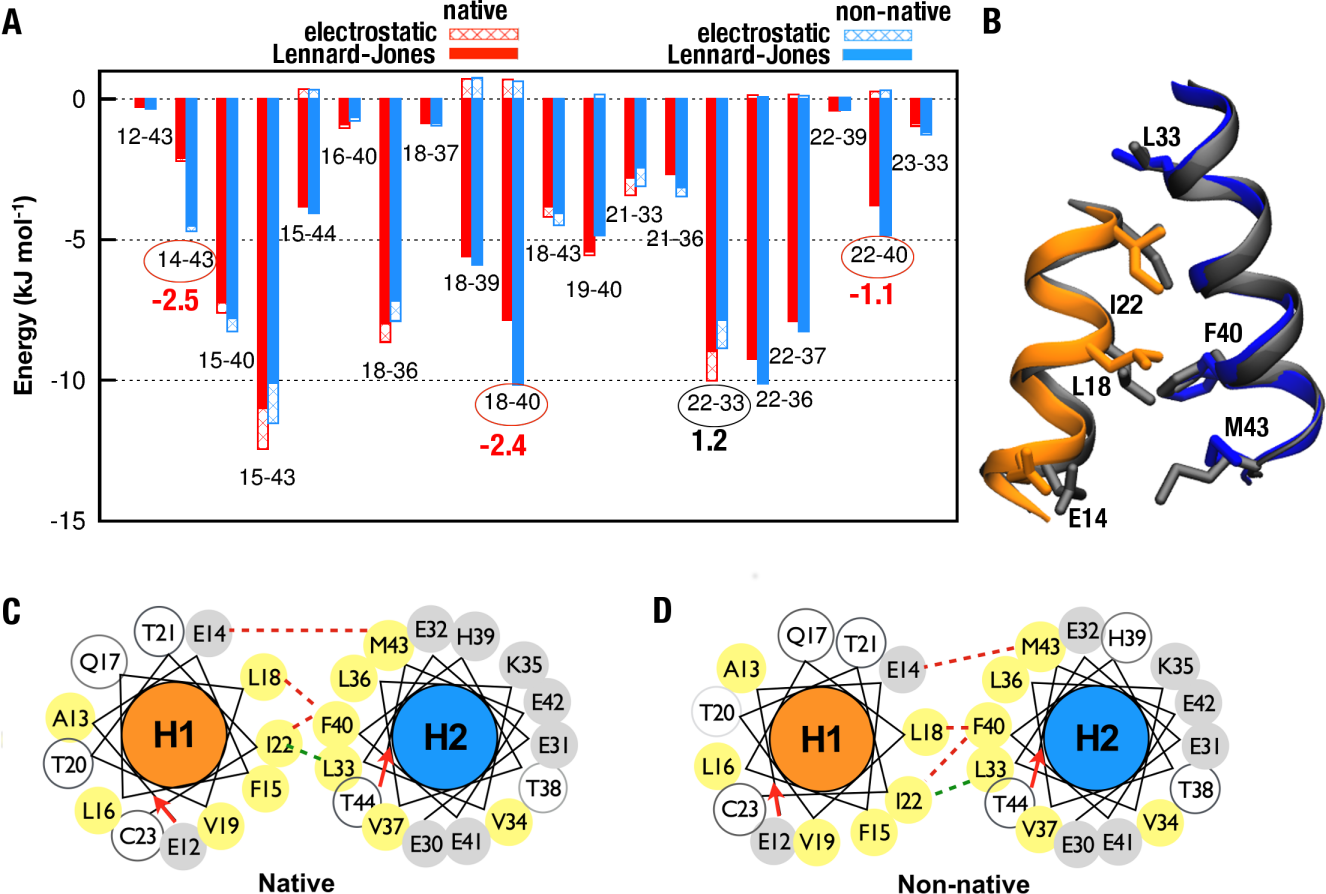
Residue-specific potential energies for Im9 H1→H2 at $d_{i}^{0}$ = 1.10 nm. (A) Lennard-Jones (filled) and electrostatic (hashed) potential energies for direct interaction of selected interfacial residue pairs from the native configuration (red) and a nonnative (blue) configuration with H1 rotated by +30°. Pairs with |*ΔE*| > 1 kJ/mol are circled (**S1 Text**). (B) Snapshot of H1 (orange) packed against H2 (blue) in the native orientation, superposed with the configuration with a nonnative +30° rotation of H1 (both helices in grey). Sidechains involved in residue pairs with |*ΔE*| > 1 kJ/mol, identified in (A), are shown as sticks. (C, D) Helical wheels show (C) native and (D) nonnative interactions between residues that contribute to the more favorable (red dashed lines) and more unfavorable (green dashed lines) component binding energies for nonnative than for native packing (see part A). As in Fig 1, amino acid residues on the helical wheels are color coded: grey for charged, yellow for nonpolar, and white for polar residues.

To better understand the driving force for nonnative H1-H2 packing, the potential energies between specific pairs of amino acid residues on the H1-H2 interface in the native orientation are compared against those in the nonnative orientation with a +30° H1 rotation. We make this comparison for helix-helix center of mass distance $d_{i}^{0}$ = 1.10 nm in both the native and non-native configurations, wherefore each pair of helices in question is in close spatial contact (Fig 4). The analysis indicates a prominent role by the more favorable Lennard-Jones interactions between interfacial residue pairs Glu14-Met43, Leu18-Phe40, and Ile22-Phe40 in favoring the nonnative packing, whereas electrostatic interactions between these residue pairs are of similar strengths for the native and nonnative packing orientations. In contrast, the interaction between Ile22 and Leu33 favors native packing, but its effect is more than compensated by the aforementioned multiple residue-residue interactions that drive nonnative packing such that a +30° rotation of H1 is favored over the native orientation for H1-H2 packing in isolation. It is noteworthy, however, that while these residue-residue energetic effects can be significant individually (Fig 4) and collectively (Table 2), they are not accompanied by obvious, drastic structural changes at the level of residue-residue contacts. When contacts between residues on different helices at a helix-packing interface are identified by a commonly used proximity threshold, contact probabilities between the helices are seen to remain essentially unchanged upon a +30° H1 native-to-nonnative rotation in both the H1→H2 and H1→ H2^L^H3^L^H4^C^ systems (**S8 Fig**).

**Fig 5:**
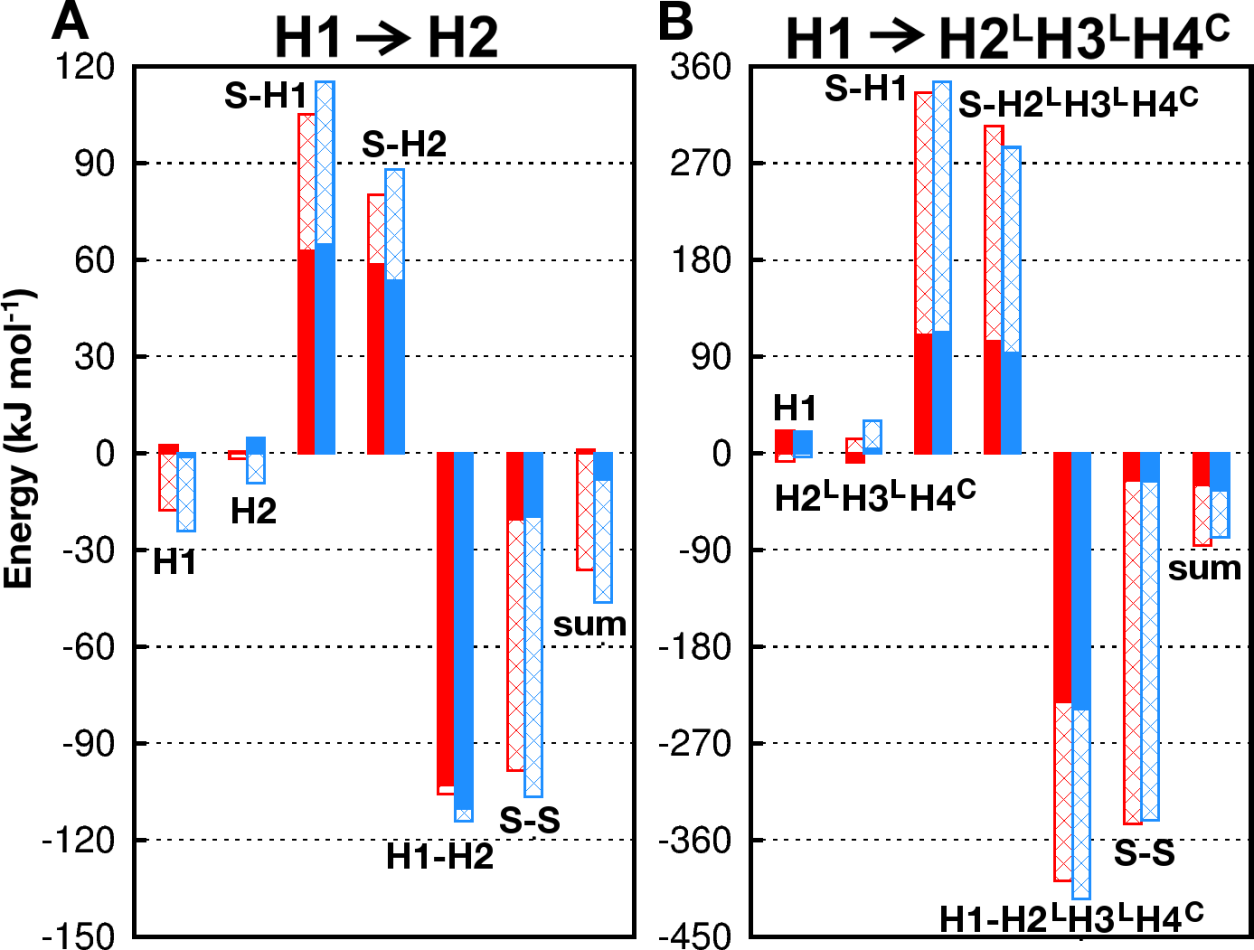
Im9 potential energies by interaction type. Average native (red) and nonnative (blue) Lennard Jones (filled) and electrostatic (hashed) energies are shown for (A) H1→H2 restrained at displacement $d_{i}^{0}$ = 1.10 nm and (B) H1→H2^L^H3^L^H4^C^ at $d_{i}^{0}$ = 1.04 nm. The notation for energy types is identical to that in Fig 4. Component energies shown here are for the interactions within H1, H2, or H2^L^H3^L^H4^C^; solvent-helix interactions (S-H1, S-H2, S-H2^L^H3^L^H4^C^); direct helix-helix interactions (H1-H2, H1-H2^L^H3^L^H4^C^); solvent-solvent interactions (S-S); and the sum of all component energies.

Seeking physical reasons for favoring native packing in H1→ H2^L^H3^L^H4^C^ but not in H1→H2, we compare the potential energies of these systems in the native and the +30° H1-rotated nonnative configurations (Fig 5). When potential energies are analyzed by the molecular species involved in the interactions, for H1→H2, solvent-protein (solvent-helix) interactions are more unfavorable with nonnative rotation of H1 by +30°, but this effect is overwhelmed by larger, favorable changes in solvent-solvent and intra- and inter-helix interactions (Fig 5A). More specifically, this nonnative H1 rotation favors inter-helix Lennard-Jones interactions (as exemplified by the three residue pairs circled in red in Fig 4A) as well as intra-helix and solvent-solvent electrostatic interactions (Fig 5A), netting an overall favorable (more negative) potential energy for the nonnative orientation (Fig 5A, “sum”). In contrast, the corresponding analysis for H1→H2^L^H3^L^H4^C^ yields a set of average potential energies that favors the native state overall (Fig 5B, native “sum” more negative than nonnative). This potential energy (enthalpic) trend is consistent with the above PMF/binding free energy prediction that the native orientation is favored for H1→H2^L^H3^L^H4^C^ (Table 2), though entropic effects may make additional contribution to the stability of native packing of H1 against H2^L^H3^L^H4^C^ (see below). Because nonnative +30° H1 rotation has opposite effects on intra-H2 (Fig 5A) versus intra-H2^L^H3^L^H4^C^ (Fig 5B) Coulomb energies, one of the reasons for disfavoring nonnative +30° H1 rotation in H1→H2^L^H3^L^H4^C^ is that this rotation of H1 induces energetic strain within H2^L^H3^L^H4^C^, resulting in a destabilizing increase in intra-H2^L^H3^L^H4^C^ Coulomb energy collectively, whereas the same +30° H1 rotation leads to an overall stabilizing decrease in intra-H2 Coulomb energy. The atomic basis of this difference remains to be analyzed.

### Entropic stabilization of native packing

**Fig 6:**
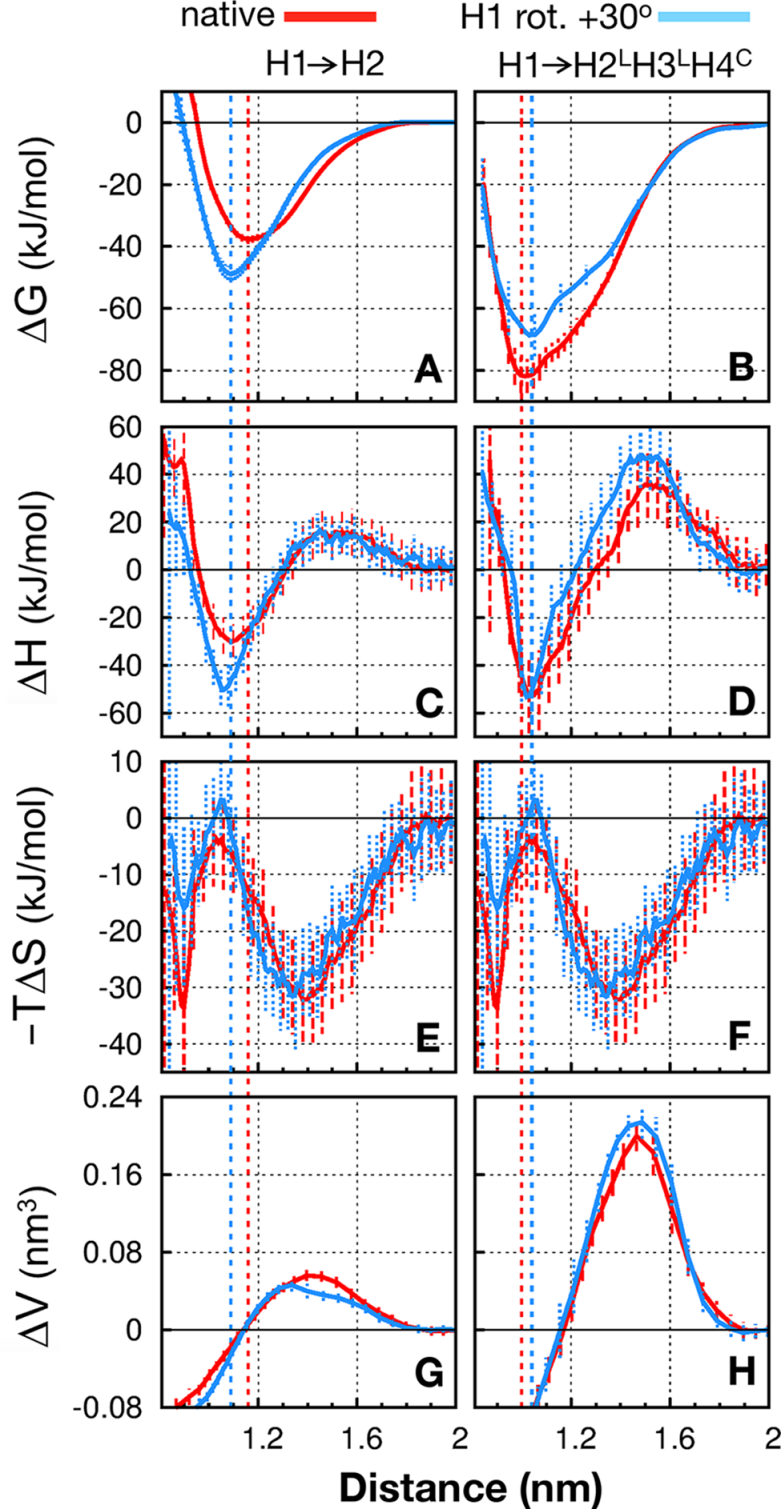
Energetic profiles of Im9 H1 binding. Change in free energy (PMF, Δ*G*), its enthalpic (Δ*H*) and entropic (−*T*Δ*S*) components, and system volume (Δ*V*) for H1 binding to other molecular fragments at 300 K as a function of the displacement, *d* (distance), between H1 and the opposing helical bundle: H1→H2 (A, C, E, G) and H1→H2^L^H3^L^H4^C^ (B, D, F, H). Simulation data are shown for the native orientation (red curves) and the nonnative orientation with +30° rotation of H1 (blue curves). Vertical dashed lines mark the positions of the native (red) and nonnative (blue) PMF minima. Error bars show standard deviations of the mean estimated by block averaging (**S1 Text**).

To gain further insight into the differential effects of H2 and H2^L^H3^L^H4^C^ on the favorability of the native orientation upon H1 binding, we resolve the distance-dependent H1→H2 and H1→H2^L^H3^L^H4^C^ PMFs (Fig 6A and 6B, respectively) into their enthalpic (Fig 6C and 6D) and entropic (Fig 6E and 6F) components. Since the backbones of the helical elements in our simulation systems are restrained to be essentially rigid, the entropic contributions computed here originate almost exclusively from the water solvent and sidechain degrees of freedom, whereas contributions from mainchain conformational entropy are negligible in comparison. Despite sampling uncertainties, several likely trends can be quite clearly discerned: For H1→H2, the lower PMF (Δ*G*) minimum for the nonnative orientation (Fig 6A) is driven by enthalpy (lower Δ*H* minimum for +30° H1 rotation than for native in Fig 6C). This effect is partially, but not completely, compensated by the entropic component of the free energy, ‒*T*Δ*G*. The latter is seen favoring native packing in Fig 6E (red curve below blue curve at distance marked by vertical blue dashed line), although the differences are largely within error bars. Entropy has a similar effect on H1→H2^L^H3^L^H4^C^ in stabilizing native packing (Fig 6F). In this case however, unlike H1→H2, enthalpy is also favorable (though only slightly) to the native state (Fig 6D, see also Fig 5B), thus the entropic and enthalpic effects reinforce each other, yielding a Δ*G* favorable to native packing for H1→H2^L^H3^L^H4^C^ (Fig 6B). It should be noted that the trends of entropic stabilization seen here in Fig 6 are similar to those exhibited by a pair of poly-alanine or poly-leucine helices [29]. In both cases, the entropic trends are likely manifestations of the well-recognized solvent-entropic origin of hydrophobic interactions at ambient temperatures.

### Enthalpic and volume barriers in helix-helix binding

Every helix-helix association in Fig 6 entails an enthalpic barrier at separation ≈1.5 nm (Fig 6C and 6D). As implied by the absence of PMF barriers at these positions (Fig 6A and 6B), the enthalpic barriers here are compensated by a larger decrease in entropic free energy at the same positions (Fig 6E and 6F). Further examples of enthalpic barriers and entropic compensations are provided in **S2 Fig**. These results are consistent with burial of hydrophobic surfaces being concomitant with increase in solvent (water) entropy at room temperature and the idea that enthalpic barriers to protein folding [20, 29, 49, 50] may arise largely from steric dewetting [29]. Because steric dewetting creates voids (between the approaching helices in the present cases; **S9 Fig**), it leads to volume barriers [29] such as those seen in Fig 6G and 6H. As has been discussed, such volume barriers probably amount to part of the activation volume of protein folding [51, 52]. For the systems studied in Fig 6, it is not surprising that the enthalpic and volume barriers are higher for H1→H2^L^H3^L^H4^C^ than for H1→H2 because the former binding process buries a significantly larger protein surface area. Therefore, we expect a larger transient void volume between the approaching helices before close packing is achieved for H1→H2^L^H3^L^H4^C^ than for H1→H2. It is interesting to note that, perhaps because void volumes are largely a consequence of geometry and less of energetics, the volume barrier heights in Fig 6G and 6H are essentially insensitive to the difference between native and nonnative packing.

## Discussion

**Fig 7:**
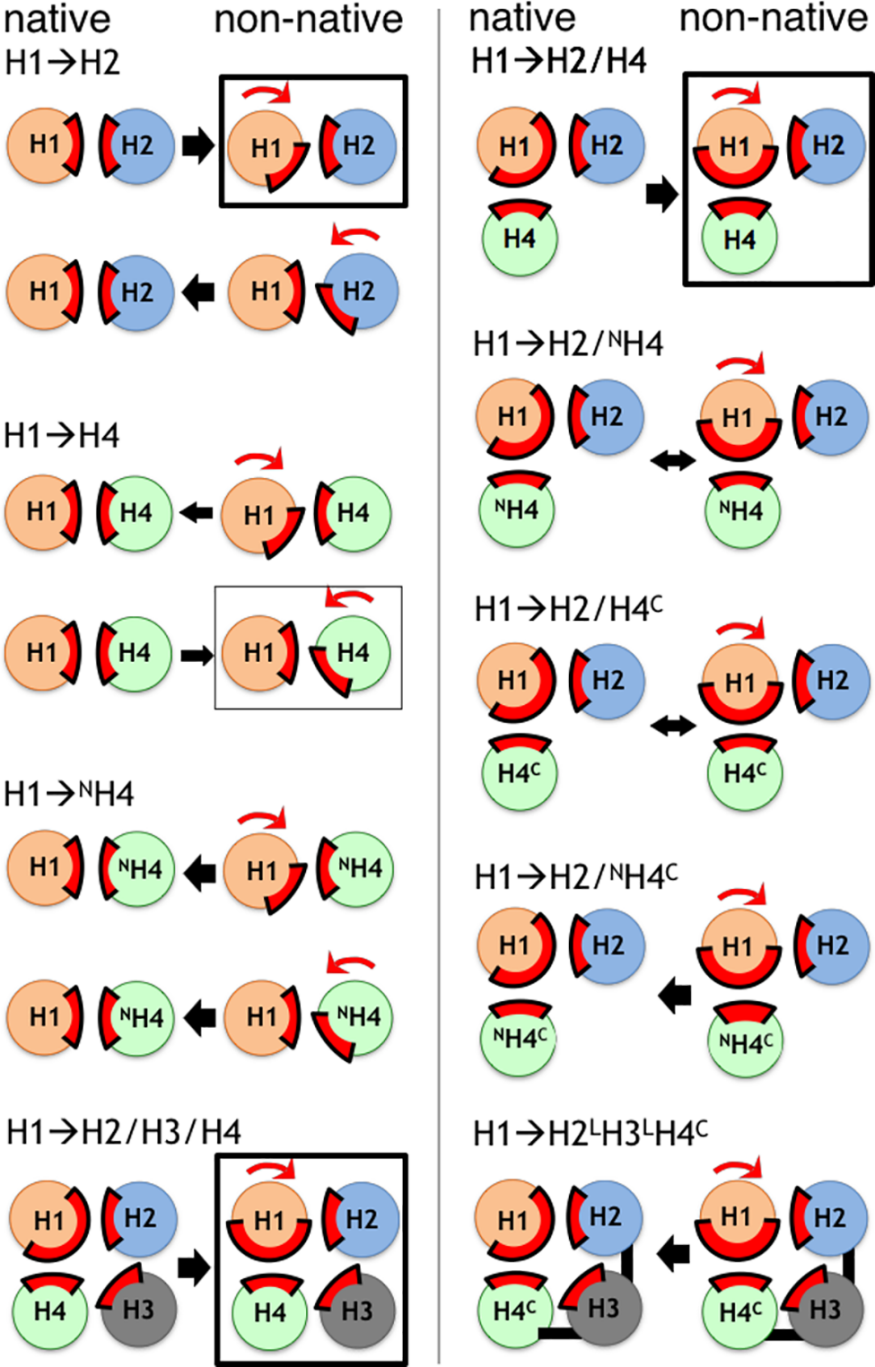
Schematics of relative preference for native versus nonnative binding in Im9 fragments. System identifiers are defined in Table 1. Helices are depicted as circles, covalent linkages are indicated by black bars, and the positions of native helix-helix interfaces are highlighted by red arcs. For each system, the configurations on the left and right are native and nonnative, respectively, with the nonnative rotation indicated by a red arrow. Black arrows point toward the orientation with a more favorable binding free energy, with bi-directional arrows indicating energetic equivalence and arrow thickness representing free energy differences with absolute values that are mild (≤ 6kJ/mol) or significant (≥ 10 kJ/mol). Black or grey boxes enclose, respectively, nonnative packing configurations that are significantly or mildly favored over native packing.

To recapitulate, we have conducted a systematic analysis of the relative stability of native versus nonnative packing of helices in the Im9 protein as a means to address the physical basis of biomolecular recognition. These results are summarized schematically in Fig 7: Relative to native packing, three nonnative configurations (H1→H2, H1→H2/H4, and H1→H2/H3/H4, each with H1 rotated) are significantly stabilized whereas one other nonnative packing orientation (H1→H4 with H4 rotated) is mildly stabilized. Other Im9 systems that we have simulated either favor the native configuration or essentially do not discriminate between native and nonnative packing. As emphasized at the outset, our method is designed to characterize packing frustration of constrained, locally native protein substructures by varying the orientation between interacting substructures that are rigid by construction, viz., the secondary structure (main-chain conformation) of each of the helices is essentially fixed. It follows that while our substantial computational effort has succeeded in gaining structurally and energetically high-resolution information about frustration that is novel and complementary to that obtained from our previous coarse-grained chain model study of Im7 and Im9 [45], the present investigation—unlike our coarse-grained modeling [45]—cannot by itself address certain general questions regarding folding pathways such as the viability of nucleation-condensation mechanisms [53] because backbone conformational freedom is not treated. For the same reason, the present method does not tackle frustration involving disordered, flexible main-chain segments that may adopt locally nonnative conformations. A notable example in this regard is the second helix of Im7. Among the four respective helices in Im7 and Im9, the amino acid sequence of the second helix varies the most between the homologs [30]. The second Im7 helix has been identified as a part of the protein which is disordered and participates in nonnative interactions that stabilize a kinetically trapped folding intermediate during the process of non-two-state folding of Im7 [45]. However, revealingly, the significant role of a disordered H2 in frustrating Im7 folding is not reflected by its behavior as an ordered helix: Unlike the H1→H2 system of Im9, the H1→H2 system of Im7 exhibits no favorable nonnative packing (**S10 Fig**). This finding underscores the importance of disordered conformations to frustration in globular protein folding, an effect that the present analysis has not addressed. From a broader perspective, such effects have to be even more critical for molecular recognition among intrinsically disordered proteins [54, 55].

Notwithstanding aforementioned limitations of the present approach, several important lessons can already be learned from our extensive computational investigation. First, a majority of the helical systems that we consider favor native packing, indicating that the Im9 amino acid sequence encodes a sufficiently strong native bias such that the native structure can be recognized by the folding protein. Second, frustration exists, manifested most notably by—but not necessarily limited to—the significantly stabilized nonnative H1-H2 packing. Although the conformational space accessible to an 86-residue polypeptide is vast compared to what is accessible via contemporary simulation and thus our ability to identify all possible sources of frustration is limited, the systematic approach taken in the present study does pinpoint one class of frustrated configurations. Third, the native fold is favored overall despite frustration, at least within the class of configurations we tested, because nonnative H1-H2 packing is destabilized when other parts of the protein, especially H4 and its flanking residues, are involved in the interaction.

A logical inference from our results is that favorable nonnative interactions can be largely suppressed during Im9 folding by favoring trajectories that assemble H1 and H2 not in isolation but only in the presence of H4 plus flanking residues. Such preference would help avoid kinetic traps to facilitate known two-state folding behaviors of Im9 [32, 36]. This expectation is consistent with the Im9 folding mechanism deduced from experimental phi-values (Φ_F_) by Radford and coworkers, who determined that residues in H2 have the lowest Φ_F_-values among H1, H2, and H4; but Φ_F_-values are higher for the hydrophobic residues in H1 and H4. This and other findings led them to conclude that the H1-H4 interface “is the most structured region in the transition state ensemble”, and that the native configuration of H1, H2, and H4 is partially formed in the transition state whereas H3 is formed after the rate-limiting step [32]. Since our simulation results also suggest that H1-H2 interactions should be weaker than those between H1-H4 to minimize kinetic trapping, our data offer a physical rationale as to why the Im9 folding pathways might have evolved.

A general theoretical formalism due to Wolynes and coworkers provides quantitative estimates of local frustration [3, 42, 56]. Of relevance here is their configurational frustration index, which quantifies the likelihood of a pair of residues that are in contact in a protein’s native structure to be engaged in favorable nonnative interactions in alternate conformations. Their web-based “Protein Frustratometer” algorithm [56] predicts a high configurational frustration region in Im9 encompassing residues 25–38, which overlaps substantially with H2 (residues 30–44, Table 1). In contrast, H1 and H4 are predicted to be situated in lower configurational frustration regions on average (**S11 Fig**). These predictions are consistent with, and therefore lend further support to the aforementioned perspective emerging from our simulation results. It is noteworthy, however, that the Frustratometer-computed configurational frustration *F*^*c*^ of Im7 is not noticeably higher on average than that of Im9 (**S11 Fig**), notwithstanding the fact that folding is significantly more frustrated for Im7 than for Im9 experimentally [30–40]. In particular, while the predicted frustration of H4 is higher for Im7 than for Im9 (which is consistent with H4’s involvement in nonnative interactions with H2 in Im7 folding), the predicted configurational frustration of H2 of Im7 is similar to, or even slightly lower than that of Im9. It would be instructive to investigate whether this apparent inability of the algorithm to clearly delineate the key experimental difference in Im7 and Im9 folding kinetics is because the decoy inter-residue contact distances used to compute configurational frustration *F*^*c*^ [56] are insufficient to fully capture the conformational possibilities of a disordered H2 that make strong nonnative interactions in Im7 possible [45]. Intuitively, this limitation might be similar or even related to the impossibility of discerning Im7 frustration from the packing of fully formed H1 and H2 alone (**S10 Fig**) despite the fact that many of the favorable nonnative interactions in Im7 folding are between residues in H1 and H2. This question deserves further attention.

Owing to the high computational cost of the present approach, applications have been confined to the commonly used OPLS-AA/L force field. While useful insights are gained as reported above, it should be noted that current molecular dynamics force fields can be limited in their ability to accurately model disordered protein states (reviewed in [57, 58]) and to capture subtle effects such as conformational switches [59]. It is important, and would be instructive, to assess how discrimination against nonnative interactions is affected by ongoing efforts to improve current force fields [57, 58]. Much work remains to be done before the physical basis of biomolecular recognition can be fully deciphered.

## Methods

We use molecular dynamics (MD) simulations to systematically study helix packing in Im9 (PDB ID: 1IMQ [47]) by constructing pairs of various Im9 fragments (bundles) comprising one or more helices (Fig 1 and Table 1) and computing their PMFs of association (Figs 2–4). A more limited set of Im7 bundles is also studied for comparison. Helical residues (Table 1) are defined by DSSP [48]. Helical rotations with positive and negative angles indicate clockwise and counter-clockwise angular displacements, respectively, around the helix’s long axis in the N- to C-terminal direction (Fig 1) relative to the native orientation. Positive and negative changes in helix-helix H1-H2 crossing angles are, respectively, rigid rotations of H1 in the clockwise and counter-clockwise directions with respect to the vector directed from the center of mass of H1 to the center of mass of H2, the angular changes being relative to the native H1-H2 crossing angle.

### Umbrella sampling with virtual replica exchange (US-VREX)

Umbrella sampling (US) [60] simulations are employed to quantify the extent to which the residues in two pre-folded regions of the protein are sufficient to drive native-like association. Specifically, we compute orientation-specific free energies for the binding of H1 to a systematic selection of helices from other parts of the protein with and without connecting loops. To enhance computational tractability, the latter helical bundles are prevented from unfolding or changing their relative orientations by imposing harmonic restraints on the positions of all C_α_ atoms with force constants of 1000 kJ/mol/nm^2^. Unfolding of H1 is disallowed by C_α_ position restraints that are enforced only in the Cartesian **y** and **z** dimensions, using the same force constant. The US order parameter is the magnitude of the Cartesian **x** component of the vector connecting the centers of mass of C_α_ atoms in the two bundles. This linear displacement, *d*, is harmonically restrained at a specified target value, $d_{i}^{0}$, in each umbrella *i*, with a force constant of 2000 kJ/mol/nm^2^. For each system, 39 umbrellas span 0.7 nm ≤ $d_{i}^{0}$ ≤ 2.6 nm in 0.05 nm increments. To further enhance the rate of convergence in these US simulations, we allow equilibrium exchange of umbrellas using the virtual replica exchange (VREX) approach [61, 62]. Further details of the US-VREX approach are provided in **S1 Text**. US-VREX simulations are conducted for the H1→H2, H1→H4, H1→^N^H4, H1→H2/H4, H1→H2/^N^H4, H1→H2/H4^C^, H1→H2/^N^H4^C^, H1→H2/H3/H4, and H1→H2^L^H3^L^H4^C^ systems of Im9 (Table 1), where the arrow separates the two interacting fragments (bundles) under consideration. The H1→H2 system of Im7 is also simulated using the same method. The two bundles in any given system are on equal footing because their association with each other is mutual. The arrow in our notation serves merely to indicate their spatial association without regard to the arrow’s direction. Each system is simulated for 100 ns/umbrella, except for H1→H2 and H1→H2^L^H3^L^H4^C^ in the native orientation and with nonnative H1 rotation by +30°, which are simulated for 500 ns/umbrella. In total, these US-VREX simulations comprise >300 μs of simulated time. Despite the application of position restraints to prevent the helices from unfolding or changing their relative orientations during PMF computations, the rotation angle of helices varies within ±10° of the target packing angle. We identify the simulated systems by the angles to which they are targeted.

### Single-chain simulations

The H1^L^H2 system comprising H1, H2, and their connecting loop is simulated from the native [47] and twenty different nonnative initial conformations generated by removing inter-helical loop residues 24–29, rotating H1 or H2 about its long axis by ±10°, ±20°, ±30°, ±40° and ±50°, and then modeling loop residues using the prediction program Loopy [63]. Secondary structure is maintained while allowing changes in helical rotation and separation by applying intra-helical distance restraints on all backbone atom pairs with force constants of 1000 kJ/mol/nm^2^. Each simulation covers 1 μs, with the first 125 ns discarded in subsequent analysis.

### Simulation protocol

MD simulations are conducted with version 4.5.5 of the GROMACS simulation package [64]. The water model is TIP3P [65]. Protein is modeled by the OPLS-AA/L parameters [66, 67]. Simulation systems are neutralized and excess NaCl is added at 0.4 M, mimicking experimental conditions [31, 68]. Water molecules are rigidified with SETTLE [69] and protein bond-lengths are constrained with P-LINCS [70]. Lennard-Jones interactions are evaluated using a group-based cutoff and truncated at 1 nm without a smoothing function. Coulomb interactions are calculated using the smooth particle-mesh Ewald method [71, 72] with a Fourier grid spacing of 0.12 nm. Simulations are in *NPT* ensembles by isotropic coupling to a Berendsen barostat [73] at 1 bar with a coupling constant of 4 ps and temperature-coupling the simulation system using velocity Langevin dynamics [74] at 300 K with a coupling constant of 1 ps. The integration time step is 2 fs. The nonbonded pair-list is updated every 20 fs. Further details are provided in **S1 Text and S1 Table**.

## Supporting information

S1 Fig Binding free energies. Values of Δ*G*_bind_ from simulations of Im9 H1→H2 (grey circles, connected by dashed lines as a guide to the eye) and H1→H2^L^H3^L^H4^C^ (black squares, connected by solid lines as a guide to the eye) at the native packing angle. Data points show the value of Δ*G*_bind_ computed from *t*‒10 to *t*+10 ns/umbrella (*i*.*e*., block averaging).
(TIF)

S2 Fig Im9 energetic profiles for H1→H2. Inter-helical PMFs (A, C, E) and distance-dependent enthalpies (B, D, F) are shown for rotation of H1 (A, B), rotation of H2 (C, D), and changing of the H1-H2 crossing angle (E, F), while leaving the backbone native configuration of the opposing helix unchanged in the spatial coordinates of the simulation system. In each plot, data for native and nonnative packing angles are shown as black and colored curves, respectively. Colors for rotation or crossing angles are listed at the top of this figure, where negative and positive angular changes are indicated, respectively, by solid and dashed lines. Error bars show standard deviations of the mean estimated by block averaging.
(TIF)

S3 FigAverage helical rotation sampled during US-VREX simulation of the H1→H2 system.Data show *actual* rotation of (A) H1 and (B) H2 for native (black curve) and nonnative orientations with H1 rotation targeted to +30° (blue curve) or −30° (red curve). Deviations between actual and targeted rotations arise from effects of many potential energy terms in the simulated system in addition to the imposed angle-restraining potential. The differences between actual and target angles shown here are relative to baselines defined by the behavior of the system at $d_{i}^{0}$ > 2.0 nm for which the interactions between the two bundles is expected to be sufficiently weak such that they may be considered to be independent.(TIF)

S4 Fig Representative structures corresponding to free energy minima for the H1→H2 system. The structures are restrained to native orientation (solid color) and nonnative orientations with H1 rotated by +30° (translucent color). Free energy minima are located at helical separation distance *d* = 1.14 nm for native orientation and *d* = 1.09 nm for nonnative orientation with H1 rotated by +30°.
(TIF)

S5 FigTwo-dimensional PMFs of the H1, H2 packing angles in H1→H2 simulations with helical rotation but no change in H1-H2 crossing angle.The format is similar to that of Fig 2 in the main text. Data are for restrained inter-helical distances, $d_{i}^{0}$, from 2.0 nm to 1.0 nm as indicated above each plot. The color scale on the right is for the *relative* free energy at any given value of $d_{i}^{0}$, but the scale does not apply across different values of $d_{i}^{0}$. White regions have no sampling.(TIF)

S6 Fig Population density maps of Im9 H1 and H2 packing angles obtained from the single-chain H1^L^H2 system. Each subplot represents an independent simulation that was initiated in either the native state (NS) or with the indicated helix rotated (R) by the specified angle.
(TIF)

S7 Fig A potential steric clash. Positive rotation of H1 brings Im9 H1 residue F15 into closer contact with C-terminal residue F83, leading to a likely steric clash if the C-terminal region retains its structure in the native state. Helices in the Im9 NMR structure (PDB ID: 1IMQ; see ref. [2] of **S1 Text**) are colored as follows: H1, orange; H2, blue; H3, black; and H4, green; whereas intervening loops and C-terminus are in grey. Enlarged view (right): F15 and F83 side chains are shown as cyan sticks in the native configuration and the F15 side chain is shown in red after rotation of H1 by +30°.
(TIF)

S8 Fig Contact probability maps of Im9 helical association. (A) Contact probabilities for H1→H2 between residues in H1 and those in H2. Here a contact is said to exist between two residues if at least two heavy atoms, one from each residue, are separated by ≤ 0.45 nm. (B, C) Corresponding contact probabilities for H1→ H2^L^H3^L^H4^C^ between residues in H2 and those in H3 and H4 (B), and between residues in H1 and those in H2, H3, and H4 (C). Color scale (top right) indicates a range from no contact (white for probability zero) to constant contact (blue for probability of one). In each of these cases (A, B, and C), results shown are for native (left panel) and nonnative rotation of H1 by +30° (right panel). For the H1→ H2^L^H3^L^H4^C^ results in (B) and (C), residues of the helices are marked by color bars to the right of each set of contact maps (H2: blue, H3: grey, H4: green).
(TIF)

S9 Fig Changes in local water density upon Im9 helix-helix binding. Colors (top scale) indicate densities that are greater (blue) or less (red) than bulk water at 300 K for a 0.4 nm slice passing through the center of mass of H1 and H2 (A, B, C) or H2^L^H3^L^H4^C^ (D, E, F). Data shown depict three representative separations between the approaching helix bundles (cf. Fig 6 of the main text): (A, D) the position corresponding to the solvent-separated enthalpy minimum at *d* = 1.90 nm, (B, E) the desolvation enthalpic barrier at *d* = 1.45 nm, and (C, F) the free energy minimum at *d* = 1.15 nm. Note that the sidechains of the approaching helix bundles are farther apart at the desolvation enthalpic barrier (B, E) than at contact (C, F). However, unlike the situation in (A, B), there is no water between the helix bundles in (B, E). Thus the total system volume is larger for (B, E) than for either (A, B) or (C, F). In other words, a volume barrier develops around *d* = 1.45 nm for both H1→H2 and H1→H2^L^H3^L^H4^C^ systems (see Fig 6G and 6H of the main text).
(TIF)

S10 Fig Im7 binding free energies for H1→H2. (A) Binding free energies, Δ*G*_bind_, for the association of H1 and H2 with native and nonnative packing angles. Nonnative configurations are generated by rotating H1 (filled black circles), or H2 (open red squares), or changing the H1-H2 crossing angle (filled blue triangles). Δ*G*_bind_ is computed by integrating the PMF over a free-energy basin as in Fig 2A and Fig 3A of the main text. (B) PMFs shown are distance-dependent free energies for the association of H1 and H2 in native (black curve) and nonnative orientations with H1 rotated by +30° (blue curve) or −30° (red curve). Standard deviations of the mean from block averaging are shown as vertical bars in (A) or shaded regions in (B). Im7 native state is from PDB 1AYI (ref. [1] of **S1 Text**), with H1 and H2 comprising residues 12–26 and 32–45, respectively, as determined by DSSP (ref. [20] of **S1 Text**).
(TIF)

S11 Fig Localized frustration computed by Protein Frustratometer 2. Data shown for (A, B) Im9 based on PDB 1IMQ (2) and (C, D) Im7 based on PDB 1AYI. (A, C) Configurational frustration index, *F*^c^, for native state contacts. Frustration increases as *F*^c^ decreases. (B, D) Stacked histograms showing proportion of contacts within 0.5 nm that are minimally frustrated (cyan; *F*^c^ >0.78), neutral (grey), or highly frustrated (red; *F*^c^ < ‒1). The positions of the four Im9/Im7 helices are shown in the same color code as in the other figures in this study. Data are computed by Protein Frustratometer 2 (ref. [19] of **S1 Text**) without electrostatics, the inclusion of which does not affect the results significantly. C-terminal Im9 residue Gly87 is omitted because it is not resolved in the 1AYI crystal structure.
(TIF)

S1 Table Unit cell dimensions and number of water molecules for Im9 simulation systems. (PDF)

S1 Text Detailed methods. (PDF)

## References

[1] Wolynes PG. As simple as can be? Nat Struct Biol. 1997;4:871–4. 9360595 10.1038/nsb1197-871

[2] Chan HS. Folding alphabets. Nat Struct Biol. 1999;6:994–6. 10542084 10.1038/14876

[3] Ferreiro DU, Komives EA, Wolynes PG. Frustration in biomolecules. Q Rev Biophys. 2015;47(3):285–363. 10.1017/S0033583514000092 PMC4256721 25225856

[4] Hemanth VV, Rao G, Gosavi S. Using the folding landscapes of proteins to understand protein function. Curr Opin Struct Biol. 2016;36:67–74. doi: 10.1016/j.sbi.2016.01.001 26812092 10.1016/j.sbi.2016.01.001

[5] Levitt M, Warshel A. Computer simulation of protein folding. Nature. 1975;253:694–8. 1167625 10.1038/253694a0

[6] Chan HS. Modeling protein density of states: Additive hydrophobic effects are insufficient for calorimetric two-state cooperativity. Proteins. 2000;40(4):543–71. 10899781 10.1002/1097-0134(20000901)40:4<543::aid-prot20>3.0.co;2-o

[7] Chan HS, Zhang Z, Wallin S, Liu Z. Cooperativity, local-nonlocal coupling, and nonnative interactions: Principles of protein folding from coarse-grained models. Annu Rev Phys Chem. 2011;62:301–26. doi: 10.1146/annurev-physchem-032210-103405 21453060 10.1146/annurev-physchem-032210-103405

[8] Lumry R, Biltonen R, Brandts JF. Validity of the “two-state” hypothesis for conformational transitions of proteins. Biopolymers. 1966;4(8):917–44. doi: 10.1002/bip.1966.360040808 5975643 10.1002/bip.1966.360040808

[9] Baker D. A surprising simplicity to protein folding. Nature. 2000;405:39–42. doi: 10.1038/35011000 10811210 10.1038/35011000

[10] Gō N. Theoretical studies of protein folding Annu Rev Biophys Bioeng. 1983;12:183–210. doi: 10.1146/annurev.bb.12.060183.001151 6347038 10.1146/annurev.bb.12.060183.001151

[11] Bryngelson JD, Wolynes PG. Spin glasses and the statistical mechanics of protein folding. Proc Natl Acad Sci USA. 1987;84(21):7524–8. 3478708 10.1073/pnas.84.21.7524 PMC299331

[12] Taketomi H, Ueda Y, Gō N. Studies on protein folding, unfolding and fluctuation by computer simulation. 1. Effect of specific amino acid sequence represented by specific inter-unit interactions. Int J Pept Protein Res. 1975;7(6):445–59. 1201909

[13] Shea JE, Onuchic JN, Brooks CL, 3rd. Exploring the origins of topological frustration: Design of a minimally frustrated model of fragment B of protein A. Proc Natl Acad Sci USA. 1999;96(22):12512–7. 10535953 10.1073/pnas.96.22.12512 PMC22965

[14] Clementi C, Nymeyer H, Onuchic JN. Topological and energetic factors: What determines the structural details of the transition state ensemble and "en-route" intermediates for protein folding? An investigation for small globular proteins. J Mol Biol 2000;298(5):937–53. doi: 10.1006/jmbi.2000.3693 10801360 10.1006/jmbi.2000.3693

[15] Zhang Z, Chan HS. Competition between native topology and nonnative interactions in simple and complex folding kinetics of natural and designed proteins. Proc Natl Acad Sci USA 2010;107(7):2920–5. doi: 10.1073/pnas.0911844107 20133730 10.1073/pnas.0911844107 PMC2840274

[16] Chen T, Song J, Chan HS. Theoretical perspectives on nonnative interactions and intrinsic disorder in protein folding and binding. Curr Opin Struct Biol. 2015;30:32–42. doi: 10.1016/j.sbi.2014.12.002 25544254 10.1016/j.sbi.2014.12.002

[17] Sikosek T, Krobath H, Chan HS. Theoretical insights into the biophysics of protein bi-stability and evolutionary switches. PLoS Comput Biol. 2016;12(6):e1004960 doi: 10.1371/journal.pcbi.1004960 27253392 10.1371/journal.pcbi.1004960 PMC4890782

[18] Levy Y, Onuchic JN. Water mediation in protein folding and molecular recognition. Annu Rev Biophys Biomol Struct. 2006;35:389–415. doi: 10.1146/annurev.biophys.35.040405.102134 16689642 10.1146/annurev.biophys.35.040405.102134

[19] Cheung MS, García AE, Onuchic JN. Protein folding mediated by solvation: water expulsion and formation of the hydrophobic core occur after the structural collapse. Proc Natl Acad Sci. 2002;99(2):685–90. doi: 10.1073/pnas.022387699 11805324 10.1073/pnas.022387699 PMC117366

[20] Chen T, Chan HS. Effects of desolvation barriers and sidechains on local-nonlocal coupling and chevron behaviors in coarse-grained models of protein folding. Phys Chem Chem Phys. 2014;16(14):6460–79. doi: 10.1039/c3cp54866j 24554086 10.1039/c3cp54866j

[21] Lindorff-Larsen K, Piana S, Dror RO, Shaw DE. How fast-folding proteins fold. Science. 2011;334(6055):517–20. doi: 10.1126/science.1208351 22034434 10.1126/science.1208351

[22] Piana S, Klepeis JL, Shaw DE. Assessing the accuracy of physical models used in protein-folding simulations: Quantitative evidence from long molecular dynamics simulations. Curr Opin Struct Biol. 2014;24:98–105. doi: 10.1016/j.sbi.2013.12.006 24463371 10.1016/j.sbi.2013.12.006

[23] Best RB, Hummer G, Eaton WA. Native contacts determine protein folding mechanisms in atomistic simulations. Proc Natl Acad Sci USA. 2013;110(44):17874–9. doi: 10.1073/pnas.1311599110 24128758 10.1073/pnas.1311599110 PMC3816414

[24] Chung HS, Piana-Agostinetti S, Shaw DE, Eaton WA. Structural origin of slow diffusion in protein folding. Science. 2015;349(6255):1504–10. doi: 10.1126/science.aab1369 26404828 10.1126/science.aab1369 PMC6260792

[25] Skinner JJ, Yu W, Gichana EK, Baxa MC, Hinshaw JR, Freed KF, et al Benchmarking all-atom simulations using hydrogen exchange. Proc Natl Acad Sci USA 2014;111(45):15975–80. doi: 10.1073/pnas.1404213111 25349413 10.1073/pnas.1404213111 PMC4234613

[26] Best RB, Zheng W, Mittal J. Balanced protein-water interactions improve properties of disordered proteins and non-specific protein association. J Chem Theory Comput 2014;10(11):5113–24. doi: 10.1021/ct500569b 25400522 10.1021/ct500569b PMC4230380

[27] Zheng W, Borgia A, Borgia MB, Schuler B, Best RB. Empirical optimization of interactions between proteins and chemical denaturants in molecular simulations. J Chem Theory Comput 2015;11(11):5543–53. doi: 10.1021/acs.jctc.5b00778 26574341 10.1021/acs.jctc.5b00778 PMC6139257

[28] Piana S, Donchev AG, Robustelli P, Shaw DE. Water dispersion interactions strongly influence simulated structural properties of disordered protein states. J Phys Chem B 2015;119(16):5113–23. doi: 10.1021/jp508971m 25764013 10.1021/jp508971m

[29] MacCallum JL, Moghaddam MS, Chan HS, Tieleman DP. Hydrophobic association of alpha-helices, steric dewetting and enthalpic barriers to protein folding. Proc Natl Acad Sci USA 2007;104(15):6206–10. doi: 10.1073/pnas.0605859104 17404236 10.1073/pnas.0605859104 PMC1847460

[30] Li W, Dennis CA, Moore GR, James R, Kleanthous C. Protein-protein interaction specificity of Im9 for the endonuclease toxin colicin E9 defined by homologue-scanning mutagenesis. J Biol Chem. 1997;272:22253–8. 9268373 10.1074/jbc.272.35.22253

[31] Ferguson N, Capaldi AP, James R, Kleanthous C, Radford SE. Rapid folding with and without populated intermediates in the homologous four-helix proteins Im7 and Im9. J Mol Biol. 1999;286(5):1597–608. doi: 10.1006/jmbi.1998.2548 10064717 10.1006/jmbi.1998.2548

[32] Friel CT, Capaldi AP, Radford SE. Structural analysis of the rate-limiting transition states in the folding of Im7 and Im9: Similarities and differences in the folding of homologous proteins. J Mol Biol. 2003;326(1):293–305. 12547210 10.1016/s0022-2836(02)01249-4

[33] Dennis CA, Videler H, Pauptit RA, Wallis R, James R, Moore GR, et al A structural comparison of the colicin immunity proteins Im7 and Im9 gives new insights into the molecular determinants of immunity-protein specificity. Biochem J. 1998;333(1):183–91. 9639578 10.1042/bj3330183 PMC1219571

[34] Capaldi AP, Shastry MCR, Kleanthous C, Roder H, Radford SE. Ultrarapid mixing experiments reveal that Im7 folds via an on-pathway intermediate. Nat Struct Biol. 2001;8(1):68–72. 11135674 10.1038/83074

[35] Capaldi AP, Kleanthous C, Radford SE. Im7 folding mechanism: misfolding on a path to the native state. Nat Struct Biol. 2002;9(3):209–16. 11875516 10.1038/nsb757

[36] Friel CT, Beddard GS, Radford SE. Switching two-state to three-state kinetics in the helical protein Im9 via the optimisation of stabilising non-native interactions by design. J Mol Biol. 2004;342(1):261–73. doi: 10.1016/j.jmb.2004.06.076 15313622 10.1016/j.jmb.2004.06.076

[37] Morton VL, Friel CL, Allen LR, Paci E, Radford SE. The effect of increasing the stability of non-native interactions on the folding landscape of the bacterial immunity protein Im9. J Mol Biol 2007;371(2):554–68. doi: 10.1016/j.jmb.2007.05.010 17574573 10.1016/j.jmb.2007.05.010

[38] Friel CT, Smith DA, Vendruscolo M, Gsponer J, Radford SE. The mechanism of folding of Im7 reveals competition between functional and kinetic evolutionary constraints. Nat Struct Mol Biol. 2009;16(3):318–24. doi: 10.1038/nsmb.1562 19252485 10.1038/nsmb.1562 PMC2651959

[39] Chen MM, Bartlett AI, Nerenberg PS, Friel CT, Hackenberger CPR, Stultz CM, et al Perturbing the folding energy landscape of the bacterial immunity protein Im7 by site-specific N-linked glycosylation. Proc Natl Acad Sci USA. 2010;107(52):22528–33. doi: 10.1073/pnas.1015356107 21148421 10.1073/pnas.1015356107 PMC3012502

[40] Pashley CL, Morgan GJ, Kalverda AP, Thompson GS, Kleanthous C, Radford SE. Conformational properties of the unfolded state of Im7 in nondenaturing conditions. J Mol Biol. 2012;416(2):300–18. doi: 10.1016/j.jmb.2011.12.041 22226836 10.1016/j.jmb.2011.12.041 PMC3314952

[41] Paci E, Friel CT, Lindorff-Larsen K, Radford SE, Karplus M, Vendruscolo M. Comparison of the transition state ensembles for folding of Im7 and Im9 determined using all-atom molecular dynamics simulations with ϕ value restraints. Proteins. 2004;54(3):513–25. doi: 10.1002/prot.10595 14747999 10.1002/prot.10595

[42] Sutto L, Lätzer J, Hegler JA, Ferreiro DU, Wolynes PG. Consequences of localized frustration for the folding mechanism of the IM7 protein. Proc Natl Acad Sci USA. 2007;104(50):19825–30. doi: 10.1073/pnas.0709922104 18077415 10.1073/pnas.0709922104 PMC2148258

[43] Luitz MP, Zacharias M. Role of tyrosine hot-spot residues at the interface of colicin E9 and immunity protein 9: A comparative free energy simulation study. Proteins. 2013;81(3):461–8. doi: 10.1002/prot.24203 23070925 10.1002/prot.24203

[44] Sun Y, Ming D. Energetic frustrations in protein folding at residue resolution: A homologous simulation study of Im9 proteins. PLoS One. 2014;9(1):e87719 doi: 10.1371/journal.pone.0087719 24498176 10.1371/journal.pone.0087719 PMC3909201

[45] Chen T, Chan HS. Native contact density and nonnative hydrophobic effects in the folding of bacterial immunity proteins. PLoS Comput Biol 2015;11(5):e1004260 doi: 10.1371/journal.pcbi.1004260 26016652 10.1371/journal.pcbi.1004260 PMC4446218

[46] Wang F, Cazzolli G, Wintrode P, Faccioli P. Folding mechanism of proteins Im7 and Im9: Insight from all-atom simulations in implicit and explicit solvent. J Phys Chem B. 2016;120(35):9297–307. doi: 10.1021/acs.jpcb.6b05819 27532482 10.1021/acs.jpcb.6b05819

[47] Osborne MJ, Breeze AL, Lian L-Y, Reilly A, James R, Kleanthous C, et al Three-dimensional solution structure and 13C nuclear magnetic resonance assignments of the Colicin E9 immunity protein Im9. Biochemistry. 1996;35(29):9505–12. doi: 10.1021/bi960401k 8755730 10.1021/bi960401k

[48] Kabsch W, Sander C. Dictionary of protein secondary structure: pattern recognition of hydrogen-bonded and geometrical features. Biopolymers. 1983;22(12):2577–637. Epub 1983/12/01. doi: 10.1002/bip.360221211 . 6667333 10.1002/bip.360221211

[49] Liu Z, Chan HS. Desolvation is a likely origin of robust enthalpic barriers to protein folding. J Mol Biol. 2005;349(4):872–89. doi: 10.1016/j.jmb.2005.03.084 15893325 10.1016/j.jmb.2005.03.084

[50] Liu Z, Chan HS. Solvation and desolvation effects in protein folding: native flexibility, kinetic cooperativity and enthalpic barriers under isostability conditions. Phys Biol 2005;2(4):S75–S85. doi: 10.1088/1478-3975/2/4/S01 16280624 10.1088/1478-3975/2/4/S01

[51] Dias CL, Chan HS. Pressure-dependent properties of elementary hydrophobic interactions: Ramifications for activation properties of protein folding. J Phys Chem B. 2014;118:7488–509. doi: 10.1021/jp501935f 24933471 10.1021/jp501935f

[52] Krobath H, Chen T, Chan HS. Volumetric physics of polypeptide coil-helix transitions. Biochemistry. 2016;55:6269–81. doi: 10.1021/acs.biochem.6b00802 27775315 10.1021/acs.biochem.6b00802

[53] Nölting B, Agard DA. How general is the nucleation-condensation mechanism. Proteins. 2008;73:754–64. doi: 10.1002/prot.22099 18498109 10.1002/prot.22099 PMC2776727

[54] Fuxreiter M, Tompa P. Fuzzy complexes: a more stochastic view of protein function. Adv Exp Med Biol. 2012;725:1–14. doi: 10.1007/978-1-4614-0659-4_1 22399315 10.1007/978-1-4614-0659-4_1

[55] Lin Y-H, Brady JP, Forman-Kay JD, Chan HS. Charge pattern matching as a ‘fuzzy’ mode of molecular recognition for the functional phase separations of intrinsically disordered proteins. New J Phys. 2017; 19:115003 doi: 10.1088/1367-2630/aa9369

[56] Parra RG, Schafer NP, Radusky LG, Tsai M-Y, Guzovsky AB, Wolynes PG, et al Protein Frustratometer 2: A tool to localize energetic frustration in protein molecules, now with electrostatics. Nucl Acids Res. 2016;44(W1):W356–60. doi: 10.1093/nar/gkw304 27131359 10.1093/nar/gkw304 PMC4987889

[57] Best RB. Computational and theoretical advances in studies of intrinsically disordered proteins. Curr Opin Struct Biol. 2017;42:147–54. doi: 10.1016/j.sbi.2017.01.006 28259050 10.1016/j.sbi.2017.01.006

[58] Levine ZA, Shea J-E. Simulations of disordered proteins and systems with conformational heterogeneity. Curr Opin Struct Biol. 2017;43:95–103. doi: 10.1016/j.sbi.2016.11.006 27988422 10.1016/j.sbi.2016.11.006

[59] Allison JR, Bergeler M, Hansen N, van Gunsteren WF. Current computer modeling cannot explain why two highly similar sequences fold into different structures. Biochemistry. 2011;50:10965–73. doi: 10.1021/bi2015663 22082195 10.1021/bi2015663

[60] Torrie GM, Valleau JP. Nonphysical sampling distributions in Monte Carlo free-energy estimation: umbrella sampling. J Comput Phys 1977;23(2):187–99.

[61] Rauscher S, Neale C, Pomès R. Simulated tempering distributed replica sampling, virtual replica exchange, and other generalized-ensemble methods for conformational sampling. J Chem Theory Comput. 2009;5(10):2640–62. doi: 10.1021/ct900302n 26631779 10.1021/ct900302n

[62] Neale C, Madill C, Rauscher S, Pomès R. Accelerating Convergence in Molecular Dynamics Simulations of Solutes in Lipid Membranes by Conducting a Random Walk along the Bilayer Normal. J Chem Theory Comput. 2013;9(8):3686–703. doi: 10.1021/ct301005b 26584121 10.1021/ct301005b

[63] Xiang Z, Soto CS, Honig B. Evaluating conformational free energies: the colony energy and its application to the problem of loop prediction. Proc Natl Acad Sci USA. 2002;99(11):7432–7. doi: 10.1073/pnas.102179699 12032300 10.1073/pnas.102179699 PMC124248

[64] Hess B, Kutzner C, van der Spoel D, Lindahl E. GROMACS 4: algorithms for highly efficient, load-balanced, and scalable molecular simulation. J Chem Theory Comput. 2008;4(3):435–47. doi: 10.1021/ct700301q 26620784 10.1021/ct700301q

[65] Jorgensen WL, Chandrasekhar J, Madura JD, Impey RW, Klein ML. Comparison of simple potential functions for simulating liquid water. J Chem Phys 1983;79(2):926–35.

[66] Jorgensen WL, Maxwell DS, Tirado-Rives J. Development and testing of the OPLS all-atom force field on conformational energetics and properties of organic liquids. J AM CHEM SOC. 1996;118(45):11225–36. doi: 10.1021/ja9621760

[67] Kaminski GA, Friesner RA, Tirado-Rives J, Jorgensen WL. Evaluation and reparametrization of the OPLS-AA force field for proteins via comparison with accurate quantum chemical calculations on peptides. J Phys Chem B. 2001;105(28):6474–87. doi: 10.1021/jp003919d

[68] Gorski SA, Capaldi AP, Kleanthous C, Radford SE. Acidic conditions stabilise intermediates populated during the folding of Im7 and Im9. J Mol Biol. 2001;312(4):849–63. http://dx.doi.org/10.1006/jmbi.2001.5001. 11575937 10.1006/jmbi.2001.5001

[69] Miyamoto S, Kollman PA. Settle: An analytical version of the SHAKE and RATTLE algorithm for rigid water models. J Comput Chem. 1992;13(8):952–62. doi: 10.1002/jcc.540130805

[70] Hess B. P-LINCS: a parallel linear constraint solver for molecular simulation. J Chem Theory Comput. 2008;4(1):116–22. doi: 10.1021/ct700200b 26619985 10.1021/ct700200b

[71] Darden T, York D, Pedersen L. Particle mesh Ewald: An *N*•log(*N*) method for Ewald sums in large systems. J Chem Phys. 1993;98(12):10089–92

[72] Essmann U, Perera L, Berkowitz ML, Darden T, Lee H, Pedersen LG. A smooth particle mesh Ewald method. J Chem Phys. 1995;103(19):8577–93.

[73] Berendsen HJC, Postma JPM, vanGunsteren WF, DiNola A, Haak JR. Molecular dynamics with coupling to an external bath. J Chem Phys. 1984;81(8):3684–90.

[74] van Gunsteren WF, Berendsen HJC. A leap-frog algorithm for stochastic dynamics. Mol Sim. 1988;1(3):173–85.

