# Supplementary figures and images for: Molecular recognition and packing frustration in a helical protein
Source: PLoS Comput Biol. 2017 Dec 19;13(12):e1005909. doi: 10.1371/journal.pcbi.1005909 (PMC5757960; doi:10.1371/journal.pcbi.1005909)

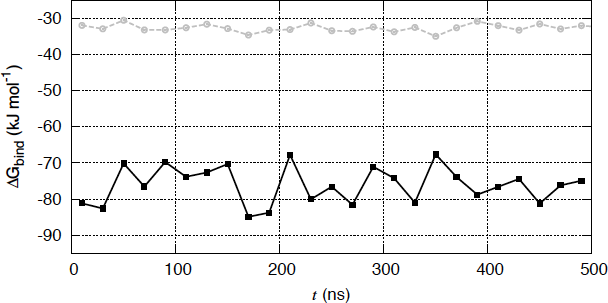

Supplement: S1 Fig — Values of ΔGbind from simulations of Im9 H1→H2 (grey circles, connected by dashed lines as a guide to the eye) and H1→H2LH3LH4C (black squares, connected by solid lines as a guide to the eye) at the native packing angle. Data points show the value of ΔGbind computed from t‒10 to t+10 ns/umbrella (i.e., block averaging). (TIF) [file pcbi.1005909.s001.tif]

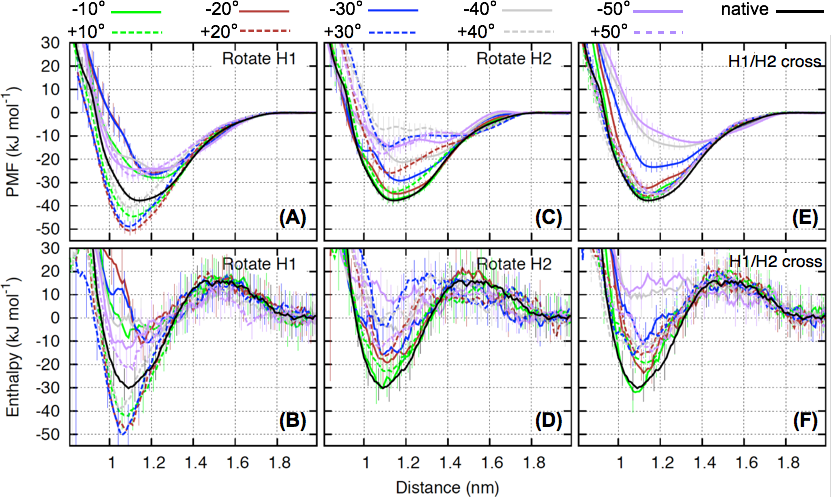

Supplement: S2 Fig — Inter-helical PMFs (A, C, E) and distance-dependent enthalpies (B, D, F) are shown for rotation of H1 (A, B), rotation of H2 (C, D), and changing of the H1-H2 crossing angle (E, F), while leaving the backbone native configuration of the opposing helix unchanged in the spatial coordinates of the simulation system. In each plot, data for native and nonnative packing angles are shown as black and colored curves, respectively. Colors for rotation or crossing angles are listed at the top of this figure, where negative and positive angular changes are indicated, respectively, by solid and dashed lines. Error bars show standard deviations of the mean estimated by block averaging. (TIF) [file pcbi.1005909.s002.tif]

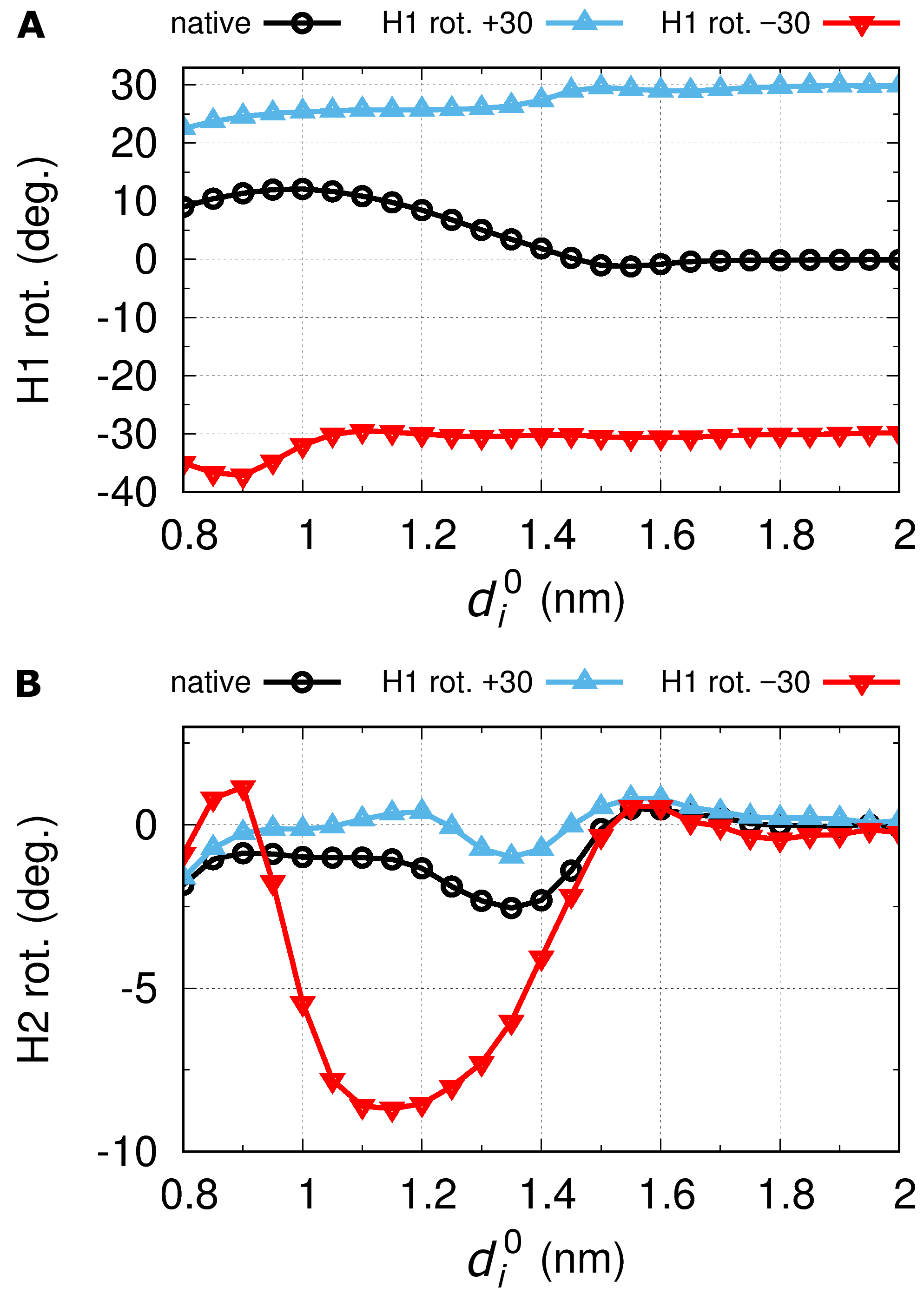

Supplement: S3 Fig — Data show actual rotation of (A) H1 and (B) H2 for native (black curve) and nonnative orientations with H1 rotation targeted to +30° (blue curve) or −30° (red curve). Deviations between actual and targeted rotations arise from effects of many potential energy terms in the simulated system in addition to the imposed angle-restraining potential. The differences between actual and target angles shown here are relative to baselines defined by the behavior of the system at di0 > 2.0 nm for which the interactions between the two bundles is expected to be sufficiently weak such that they may be considered to be independent. (TIF) [file pcbi.1005909.s003.tif]

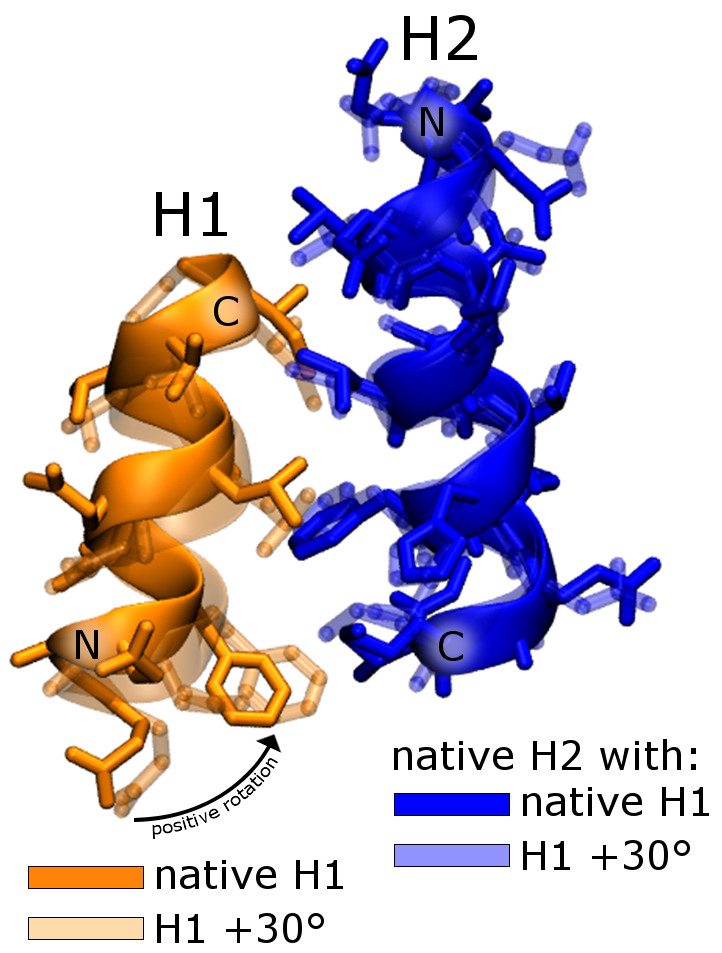

Supplement: S4 Fig — The structures are restrained to native orientation (solid color) and nonnative orientations with H1 rotated by +30° (translucent color). Free energy minima are located at helical separation distance d = 1.14 nm for native orientation and d = 1.09 nm for nonnative orientation with H1 rotated by +30°. (TIF) [file pcbi.1005909.s004.tif]

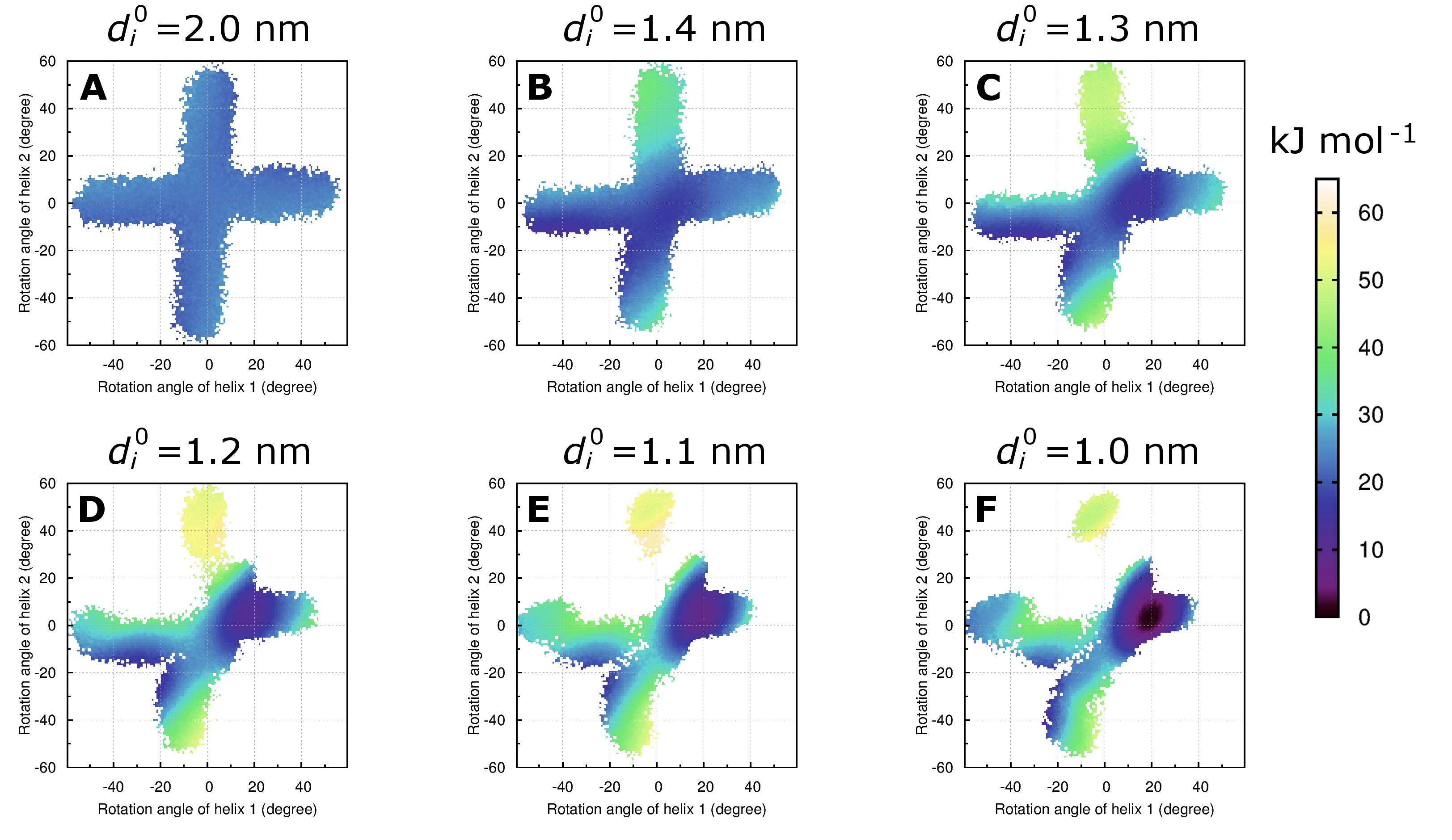

Supplement: S5 Fig — The format is similar to that of Fig 2 in the main text. Data are for restrained inter-helical distances, di0, from 2.0 nm to 1.0 nm as indicated above each plot. The color scale on the right is for the relative free energy at any given value of di0, but the scale does not apply across different values of di0. White regions have no sampling. (TIF) [file pcbi.1005909.s005.tif]

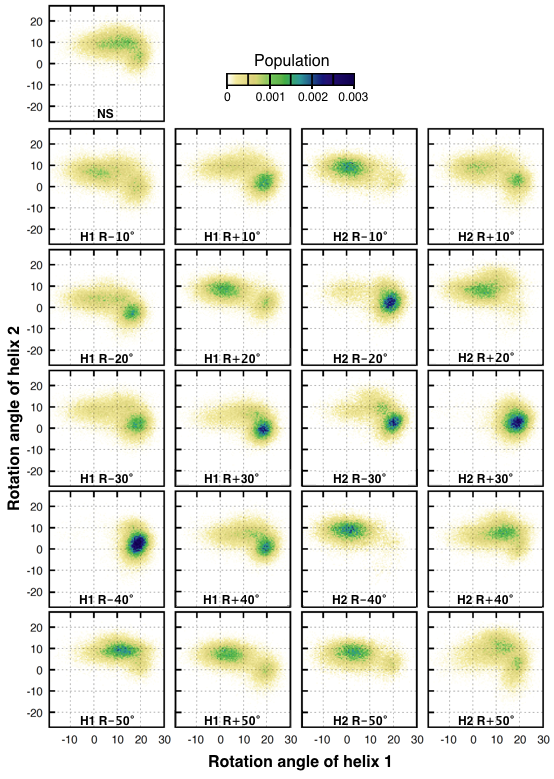

Supplement: S6 Fig — Each subplot represents an independent simulation that was initiated in either the native state (NS) or with the indicated helix rotated (R) by the specified angle. (TIF) [file pcbi.1005909.s006.tif]

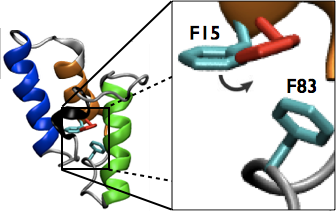

Supplement: S7 Fig — Positive rotation of H1 brings Im9 H1 residue F15 into closer contact with C-terminal residue F83, leading to a likely steric clash if the C-terminal region retains its structure in the native state. Helices in the Im9 NMR structure (PDB ID: 1IMQ; see ref. [2] of S1 Text) are colored as follows: H1, orange; H2, blue; H3, black; and H4, green; whereas intervening loops and C-terminus are in grey. Enlarged view (right): F15 and F83 side chains are shown as cyan sticks in the native configuration and the F15 side chain is shown in red after rotation of H1 by +30°. (TIF) [file pcbi.1005909.s007.tif]

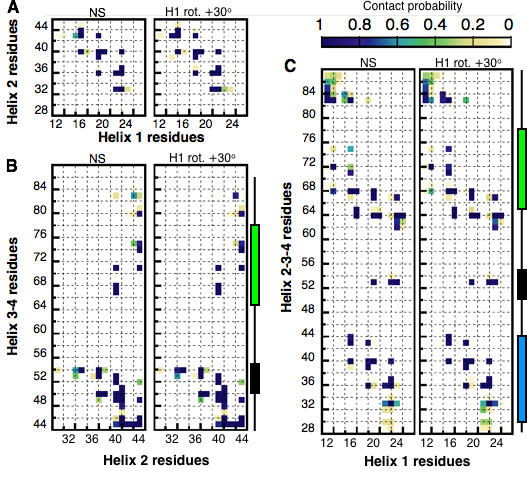

Supplement: S8 Fig — (A) Contact probabilities for H1→H2 between residues in H1 and those in H2. Here a contact is said to exist between two residues if at least two heavy atoms, one from each residue, are separated by ≤ 0.45 nm. (B, C) Corresponding contact probabilities for H1→ H2LH3LH4C between residues in H2 and those in H3 and H4 (B), and between residues in H1 and those in H2, H3, and H4 (C). Color scale (top right) indicates a range from no contact (white for probability zero) to constant contact (blue for probability of one). In each of these cases (A, B, and C), results shown are for native (left panel) and nonnative rotation of H1 by +30° (right panel). For the H1→ H2LH3LH4C results in (B) and (C), residues of the helices are marked by color bars to the right of each set of contact maps (H2: blue, H3: grey, H4: green). (TIF) [file pcbi.1005909.s008.tif]

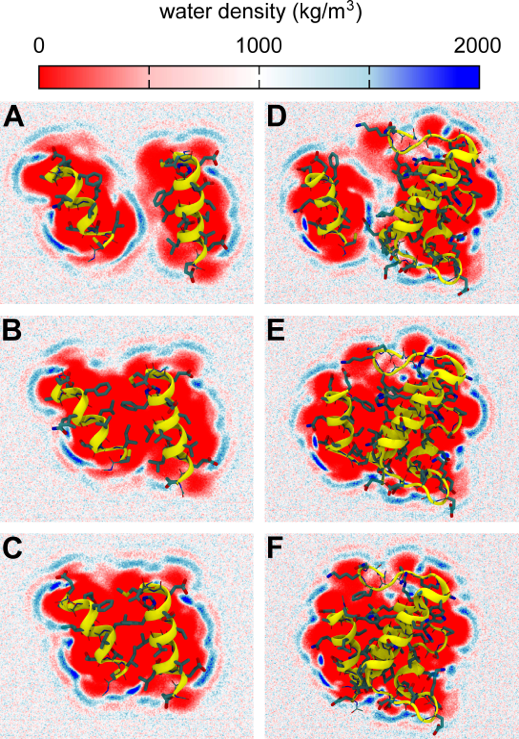

Supplement: S9 Fig — Colors (top scale) indicate densities that are greater (blue) or less (red) than bulk water at 300 K for a 0.4 nm slice passing through the center of mass of H1 and H2 (A, B, C) or H2LH3LH4C (D, E, F). Data shown depict three representative separations between the approaching helix bundles (cf. Fig 6 of the main text): (A, D) the position corresponding to the solvent-separated enthalpy minimum at d = 1.90 nm, (B, E) the desolvation enthalpic barrier at d = 1.45 nm, and (C, F) the free energy minimum at d = 1.15 nm. Note that the sidechains of the approaching helix bundles are farther apart at the desolvation enthalpic barrier (B, E) than at contact (C, F). However, unlike the situation in (A, B), there is no water between the helix bundles in (B, E). Thus the total system volume is larger for (B, E) than for either (A, B) or (C, F). In other words, a volume barrier develops around d = 1.45 nm for both H1→H2 and H1→H2LH3LH4C systems (see Fig 6G and 6H of the main text). (TIF) [file pcbi.1005909.s009.tif]

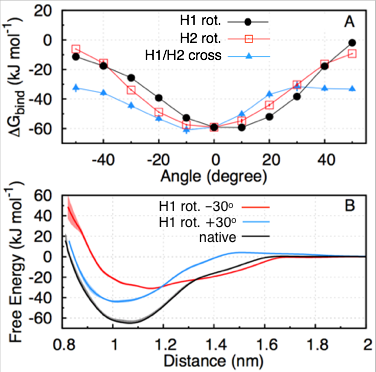

Supplement: S10 Fig — (A) Binding free energies, ΔGbind, for the association of H1 and H2 with native and nonnative packing angles. Nonnative configurations are generated by rotating H1 (filled black circles), or H2 (open red squares), or changing the H1-H2 crossing angle (filled blue triangles). ΔGbind is computed by integrating the PMF over a free-energy basin as in Fig 2A and Fig 3A of the main text. (B) PMFs shown are distance-dependent free energies for the association of H1 and H2 in native (black curve) and nonnative orientations with H1 rotated by +30° (blue curve) or −30° (red curve). Standard deviations of the mean from block averaging are shown as vertical bars in (A) or shaded regions in (B). Im7 native state is from PDB 1AYI (ref. [1] of S1 Text), with H1 and H2 comprising residues 12–26 and 32–45, respectively, as determined by DSSP (ref. [20] of S1 Text). (TIF) [file pcbi.1005909.s010.tif]

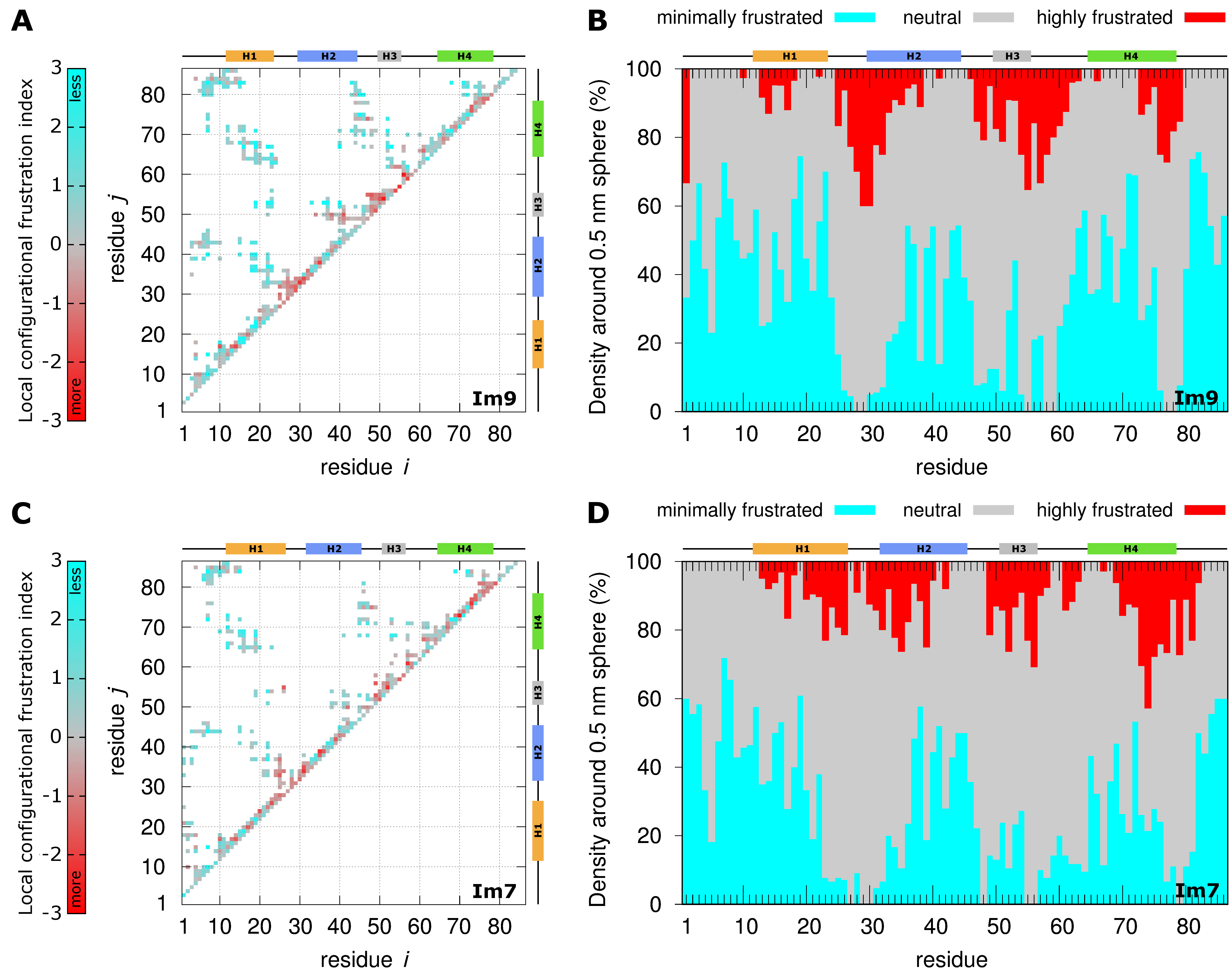

Supplement: S11 Fig — Data shown for (A, B) Im9 based on PDB 1IMQ (2) and (C, D) Im7 based on PDB 1AYI. (A, C) Configurational frustration index, Fc, for native state contacts. Frustration increases as Fc decreases. (B, D) Stacked histograms showing proportion of contacts within 0.5 nm that are minimally frustrated (cyan; Fc >0.78), neutral (grey), or highly frustrated (red; Fc < ‒1). The positions of the four Im9/Im7 helices are shown in the same color code as in the other figures in this study. Data are computed by Protein Frustratometer 2 (ref. [19] of S1 Text) without electrostatics, the inclusion of which does not affect the results significantly. C-terminal Im9 residue Gly87 is omitted because it is not resolved in the 1AYI crystal structure. (TIF) [file pcbi.1005909.s011.tif]
