# Supplementary material for: Molecular recognition and packing frustration in a helical protein
Source: PLoS Comput Biol. 2017 Dec 19;13(12):e1005909. doi: 10.1371/journal.pcbi.1005909 (PMC5757960; doi:10.1371/journal.pcbi.1005909)
Supplement: S1 Table — (PDF) [file pcbi.1005909.s012.pdf]

SUPPORTING TABLE

S1 Table. Unit cell dimensions and number of water molecules for Im9 simulation systems.

System Identifier[a]	Dimensions (nm)	No. water molecules
H1→H2	6.0 × 3.8 × 3.3	2370
H1→H4	4.7 × 3.8 × 3.3	2370
H1→ ^{N}H4	4.7 × 3.8 × 3.3	2370
H1→H2/H4	6.1 × 4.7 × 4.4	3800
H1→H2/ ^{N}H4	6.0 × 5.2 × 4.5	4200
H1→H2/H4^{C}	6.0 × 5.2 × 4.5	4200
H1→H2/ ^{N}H4^{C}	6.0 × 5.2 × 4.5	4200
H1→H2/H3/H4	6.0 × 5.2 × 4.5	4200
H1→H2$^{\mathbf{L}}$H3$^{\mathbf{L}}$H4^{C}	6.0 × 5.2 × 4.5	4200
H1$^{\mathbf{L}}$H2	4.7 × 4.5 × 4.5	2900

[a]The two interacting bundles in each system are separated by an arrow. Superscripts “N” and “C” represent, respectively, the three residues N-terminal to H4 and the eight residues C-terminal to H4. Superscript “L” represents the loop residues connecting two consecutive helices, whereas a slash between two helix-containing blocks of residues indicates that the chain segment between the blocks is not part of the bundle of interest.
