# Supplementary material for: Molecular recognition and packing frustration in a helical protein
Source: PLoS Comput Biol. 2017 Dec 19;13(12):e1005909. doi: 10.1371/journal.pcbi.1005909 (PMC5757960; doi:10.1371/journal.pcbi.1005909)
Supplement: S1 Text — (PDF) [file pcbi.1005909.s013.pdf]

Molecular Recognition and Packing Frustration in a Helical Protein

Supporting Information

Loan Huynh[a], Chris Neale[b], Régis Pomès[c,a,1] and Hue Sun Chan[a,d,1]

[a]Department of Biochemistry, University of Toronto, 1 King's College Circle, Toronto, Ontario, M5S 1A8, Canada

[b]Department of Physics, Applied Physics, and Astronomy, Rensselaer Polytechnic Institute, Troy, New York, 12180, USA

[c]Molecular Medicine, The Hospital for Sick Children, 686 Bay Street, Toronto, Ontario, M5G 0A4, Canada

[d]Department of Molecular Genetics, University of Toronto, 1 King's College Circle, Toronto, Ontario, M5S 1A8, Canada

[1]Corresponding authors: Hue Sun Chan; E-mail: chan@arrhenius.med.toronto.edu

Régis Pomès; E-mail: pomes@sickkids.ca

Supporting Information Text (S1 Text)

Detailed Methods

We provide below the details of our methodology outlined in *Methods* of the main text.

System setup. The bulk of the present simulation systems comprise fragments (also referred to as "bundles") of the *E. Coli* colicin immunity protein Im9 in explicit water. The Im9 protein has four α-helices packed around a hydrophobic core. The helices are connected by loops and attached to terminal chain segments. Though the connecting loops and termini lack secondary structure (excepting a short 3_{10}-helix at the N-terminus), they are generally well defined in experimentally determined structures [1, 2]. Starting Im9 configurations in our simulations are taken from the energy-minimized average NMR structure of Osborne *et al.* (PDB ID: 1IMQ [2]), referred to as the native state hereafter and also in the main text. This study employs nine different simulations systems for Im9, each containing different components of the protein (Table 1 of the main text). In all cases, N- and C-termini of protein fragments are acetylated and amidated, respectively. In particular, the bona-fide C-terminus of the entire protein is also capped for consistency when it is present in the system of interest.

For the H1→H2 system, a total of 31 configurations are constructed (one native and thirty nonnative, Fig.2A of the main text). Nonnative configurations are constructed by rotating helix 1 (H1) or helix 2 (H2) about its long axis by ±10°, ±20°, ±30°, ±40°, and ±50°, or by changing the H1-H2 crossing angle (*i.e.*, rotating along an axis defined by the vector that points from the center of mass of H1 to the center of mass of H2) by ±10°, ±20°, ±30°, ±40°, and ±50° relative to the native crossing angle, as prescribed in *Methods* of the main text. Operationally, configurations with a given positive (or negative) change in the H1-H2 crossing angle, $\delta\theta$, are constructed by first placing the center of mass of H1 at the origin and the center of mass of H2 at a point on the positive **x** axis of a Cartesian coordinate system such that the relative orientations of the two helices are identical to that in the native structure except allowing for linear displacements between their centers of mass. H1 is then rotated clockwise (or counter-clockwise) with respect to the positive **x** axis while holding H2 fixed such that the projection of the resulting H1 helical axis on the **yz** plane ends up subtending an angle $\delta\theta$ with its original direction on the **yz** plane. In constructing these configurations, we have taken care to ensure that these rotation angles and changes in crossing angles used in our simulations are geometrically compatible with

connection of H1 to H2 by its intervening 6-residue loop although the loop itself is not present in the simulations.

The H1→H4 system is constructed similarly, with analogous rotation of H4 in place of H2 but crossing angle changes are not considered.

For the remaining H1→^{N}H4, H1→H2/H4, H1→H2/^{N}H4, H1→H2/H4^{C}, H1→H2/^{N}H4^{C}, H1→H2/H3/H4, and H1→H2^{L}H3^{L}H4^{C} systems, only native and nonnative +30° rotations of H1 or ^{N}H4 are considered. This particular nonnative rotation is used for the following reason. Because binding free energies at +20° and +30° H1 rotation are not significantly different in the H1→H2 system (Fig. 2A of the main text), we expect a larger rotation of H1 (+30° instead of +20°) to provide binding free energy differences that are easier to interpret given our use of harmonic restraints rather than holonomic constraints to maintain helical orientations. Loop conformations from the native state are used for the H1→ H2^{L}H3^{L}H4^{C} system. Number of water molecules and initial box dimensions are indicated for all simulation systems in **S1 Table**. Simulations of H1→H2 systems for Im7 are also conducted for comparison (S10 Fig).

Umbrella sampling with exchange of umbrellas (US-VREX). Virtual replica exchange (VREX) is a generalized ensemble sampling algorithm. Similar to replica exchange (RE) [3-7], VREX comprises a set of simulations (replicas) each of which samples a target ensemble that is restrained at a different position along a globally defined order parameter. In RE, pairs of simulations are periodically subjected to a reciprocal exchange attempt based on their potential energies and their instantaneous positions along the order parameter using a Metropolis Monte Carlo criterion that preserves equilibrium populations within each ensemble. VREX improves upon the computational efficiency of RE by removing the need for synchronicity of the two simulations in the exchange attempt. This asynchronicity is accomplished by storing the instantaneous value on which exchange is based, in a list that is associated with the target sampling ensemble. These stored values are used subsequently in virtual reverse exchange attempts while evaluating the probability of a transition for a replica in a neighboring ensemble [8]. In this work, we use the approach of Neale *et al.* to conduct a random walk in helical displacement by exchanging umbrellas, i.e., umbrella sampling-VREX or US-VREX in short [9].

US-VREX simulations employ 39 umbrellas using the GROMACS pull code. Following 5,000 steps of steepest-descent energy minimization, 3 ns of molecular dynamics (MD)

simulation are performed in the *NPT* ensemble to generate initial conformations. In US-VREX simulations, the helical displacement, d, is harmonically restrained at a specified target value, d_i^0, in each umbrella i, with a force constant, k_u = 2000 kJ/mol/nm^2. Stochastic jumps in d_i^0 are attempted every 4 ps for a total of 100 ns per umbrella (5×10^4 exchange attempts per replica) or a total of 500 ns per umbrella (2.5×10^5 exchange attempts per replica). No values are stored on virtual-exchange lists [9] in the first 250 simulation segments of any replicas and no exchanges are attempted for the first 300 simulation segments. Sampled values are stored every 0.2 ps.

Free energies. The values of d sampled in US-VREX simulations are converted to PMFs by using Alan Grossfield's implementation [10] of the weighted histogram analysis method (WHAM) [11]. Accordingly, recorded values of helical separation in the range 0.7 nm ≤ d ≤ 2.6 nm are distributed among 500 histogram bins and the WHAM calculation is performed with a tolerance of 4.184×10^{-5} kJ/mol. Each resulting PMF describes the free energy as a function of separation distance, ΔG_d, from a maximal separation of d_i^0 =2.6 nm to closest (contact) interaction at d_i^0 = 0.7 nm. Each PMF is then shifted such that the average value of ΔG_d in the range 2.0 nm ≤ d ≤ 2.6 nm, denoted as ΔG_{bulk}, is equal to zero. The standard deviation of the mean values of PMFs are calculated using the block averaging technique [12] by computing PMFs from blocks of 20 ns and 120 ns, respectively, for simulations with 100 ns and 500 ns/umbrella. The binding free energy, ΔG_{bind}, used in the present analysis is obtained by a procedure of trapezoid integration of the PMF similar to one employed previously [13] over a free energy basin defined to be in the range 0.7 nm ≤ d ≤ 2.0 nm, viz.,

$$\Delta G_{\text{bind}} = -\frac{1}{\beta}\ln \int_{d=0.7\text{ nm}}^{2.0\text{ nm}} e^{-\beta \Delta G_d}\, d\left(d\right). \tag{S1}$$

Here $\beta=(k_B T)^{-1}$ where k_B is Boltzmann constant and T is absolute temperature; $d(d)$ is the differential element for the helix-helix distance d. Effectively, the quantity ΔG_{bind} is defined relative to an unbound ΔG_d = 0 reference state that has a d-value range of 1.0 nm [13]. The ΔG_{bind} so defined by Eq. S1 is not dependent upon concentration although concentration effects can be incorporated in a derived quantity of ΔG_{bind} that takes into account translational entropy of the bundles. Because the free energy cost of conformational restraints should be independent of the packing angle when the bundles are sufficiently far from one another (taken to be d > 2.0

nm in the present calculation), we may quantify the relative binding affinities of simulated systems with different compositions and/or orientations by comparing their ΔG_{bind} values without regard to the particulars of their common reference state.

To assess systematic sampling errors in our free energy computation, we use WHAM to compute free energy profiles based on data sets comprising sampling from time t to $t + \Delta t$ from each umbrella independently for Δt = 20 ns and increasing values of t. We then use visual inspection to determine the amount of initial non-equilibrium sampling (20 ns per umbrella). After discarding this initial equilibrating sampling, the uncertainty in ΔG_{bind} is estimated by its standard deviation, $\sigma_{\Delta G_{\mathrm{bind}}}$, which is calculated after dividing the remaining trajectory into three blocks (i.e., Δt = 27 and 160 ns, respectively, for the simulations with 100 and 500 ns/umbrella).

When evaluating relative free energy differences, $\Delta\Delta G_{\mathrm{bind}} = \Delta G_{\mathrm{bind}}(i) - \Delta G_{\mathrm{bind}}(j)$, for orientations i (corresponding to +30° helical rotation) and j (native; see Tables 1 and 2 of the main text), we propagate $\sigma_{\Delta G_{\mathrm{bind}}}$ to obtain $\sigma_{\Delta\Delta G} = \sqrt{\sigma_{\Delta G_{\mathrm{bind}}(i)}{}^2 + \sigma_{\Delta G_{\mathrm{bind}}(j)}{}^2}$. Probability ratios, P^{ratio}, are obtained from values of $\Delta\Delta G_{\mathrm{bind}}$ using $P^{\mathrm{ratio}} = e^{-\beta\Delta\Delta G_{\mathrm{bind}}}$.

Two-Dimensional PMFs. We provide two two-dimensional PMFs for H1→H2 and H1→H4 in Fig. 2C and Fig. 3C of the main text, respectively. During simulation, helical rotations are enforced by way of position restraints for C_α atoms (see main text *Methods*) rather than *bona-fide* helical orientation restraints. Therefore, we assume that the C_α spatial restraints can be collectively approximated by a single rotational restraint and estimate the effective force constants for helical rotation. These effective force constants are then used to combine the sampled ensembles so as to generate a PMF via WHAM. To this end, we use the sampled values of the helical rotation angle, θ, and the average sampled rotation angle, $\bar{\theta}$, to compute histograms of the deviation of the sampled rotation, $\Delta\theta = \theta - \bar{\theta}$, for d > 2.0 nm, where the PMFs are flat. The average rotation angle at d > 2.0 nm is used as a reporter on the center of restraint because of uncertainties associated with defining the helical rotation angle based on the positions of fluctuating C_α atoms. The probability histogram of $\Delta\theta$, $P_{\Delta\theta}$, is converted to a profile of the rotational free energy, $\Delta G_{\Delta\theta}$, according to $\Delta G_{\Delta\theta} = -k_B T \ln[P_{\Delta\theta}]$. A harmonic biasing potential of the form $U_{\Delta\theta} = 1/2\, k_c \left(\Delta\theta\right)^2$ is then fitted to the profile of $\Delta G_{\Delta\theta}$ obtained from our simulations to ascertain the effective force constant, k_c. Finally, we utilize the sampling from US-VREX

simulations at all combinations of restrained helical rotations, each at the value of d_i^0 corresponding to the most favorable distance for that set of restraints (determined from **S2 Fig** for the H1→H2 system and analogously for the H1→H4 system), to compute the free energy surface using Alan Grossfield's periodic implementation of the 2D-WHAM [11, 14-16] by applying a tolerance of 4.184×10^{-5} kJ/mol. In doing so, the orthogonal order parameters in the H1→H2 system have effective force constants of 0.1099, 0.1225, and 0.1709 kJ/mol/deg^2, respectively, for rotation of H1, H2, and H4.

Potential energy decomposition. We decompose potential energies in two ways to identify interactions that drive native and nonnative helical association.

First, we compute potential energy contributions from direct interactions between pairs of residues in opposing helical bundles. These energies, shown in Fig. 4A of the main text for native and nonnative packing with +30° rotation of H1, represent the average sum of inter-residue Lennard-Jones (LJ) and electrostatic interactions at d_i^0 = 1.10 nm (data from the first 10 ns/umbrella are excluded from the average). In this analysis, we only consider pairs of residues with average minimum heavy-atom contact distances ≤ 0.45 nm. This cutoff distance corresponds to the first minimum in the radial distribution function. Long-range electrostatic Particle Mesh Ewald (PME) contributions are not included. When comparing potential energies between native and nonnative helical arrangements, differences in potential energies are calculated by subtracting the potential energies of the native orientation (*i.e.* the reference state) from those of the nonnative orientation.

Second, we compute potential energy contributions from interactions between different molecular species. These energies are shown in Fig. 5 of the main text. Here, LJ and short-range electrostatic (i.e., without PME) interactions are decomposed for e.g., H1-H2 or H1-solvent interactions, etc.

Free energy decomposition. Decompositions of changes in free energy, ΔG, into enthalpic, ΔH, and entropic, $-T\Delta S$, components are accomplished by equating ΔH to the average change in internal energy, ΔU, which is the change in the sum of potential and kinetic energies, plus the $P\Delta V$ contribution, viz., $\Delta H = \Delta U + P\Delta V$, and calculating $-T\Delta S$ according to $-T\Delta S = \Delta G - \Delta H$. Every Δ-quantity is the value of the given quantity at distance d minus the average of the

quantity in the region 2.0 nm ≤ d ≤ 2.6 nm that we use to define the ΔG baseline (see above). Volume V is obtained by averaging over NPT simulation box sizes and P = 1 bar, as has been specified in the main text. This analysis is applied to H1→H2 and H1→H2^{L}H3^{L}H4^{C} in Fig. 6 of the main text and ***S2* Fig**. While statistical error can probably be reduced by computing the entropic component via the temperature-derivative of the entropy, i.e., by conducting simulations at multiple temperatures, average internal energies should provide adequate results [17] for our present purposes.

Solvent-accessible surface area. Solvent accessible surface area is computed using a GROMACS analysis tool that implements the algorithm outlined by Eisenhaber *et al* [18]. For terminological simplicity, it is referred to as “surface area” in the main text.

SUPPORTING TABLE

S1 Table. Unit cell dimensions and number of water molecules for Im9 simulation systems.

System Identifier[a]	Dimensions (nm)	No. water molecules
H1→H2	6.0 × 3.8 × 3.3	2370
H1→H4	4.7 × 3.8 × 3.3	2370
H1→ ^{N}H4	4.7 × 3.8 × 3.3	2370
H1→H2/H4	6.1 × 4.7 × 4.4	3800
H1→H2/ ^{N}H4	6.0 × 5.2 × 4.5	4200
H1→H2/H4^{C}	6.0 × 5.2 × 4.5	4200
H1→H2/ ^{N}H4^{C}	6.0 × 5.2 × 4.5	4200
H1→H2/H3/H4	6.0 × 5.2 × 4.5	4200
H1→H2$^{\mathbf{L}}$H3$^{\mathbf{L}}$H4^{C}	6.0 × 5.2 × 4.5	4200
H1$^{\mathbf{L}}$H2	4.7 × 4.5 × 4.5	2900

[a]The two interacting bundles in each system are separated by an arrow. Superscripts “N” and “C” represent, respectively, the three residues N-terminal to H4 and the eight residues C-terminal to H4. Superscript “L” represents the loop residues connecting two consecutive helices, whereas a slash between two helix-containing blocks of residues indicates that the chain segment between the blocks is not part of the bundle of interest.

SUPPORTING FIGURES

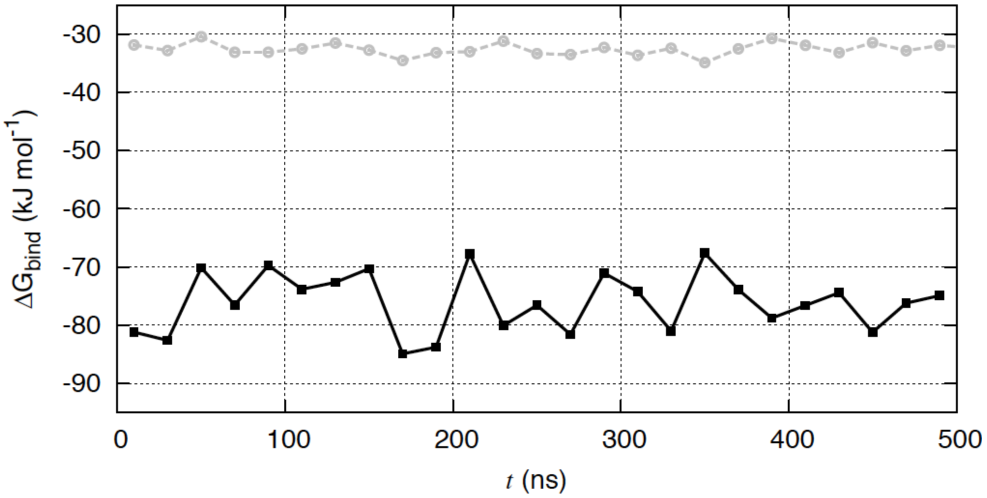

S1 Fig. Binding free energies. Values of ΔG_{bind} from simulations of Im9 H1→H2 (grey circles, connected by dashed lines as a guide to the eye) and H1→H2^{L}H3^{L}H4^{C} (black squares, connected by solid lines as a guide to the eye) at the native packing angle. Data points show the value of ΔG_{bind} computed from *t*–10 to *t*+10 ns/umbrella (*i.e.*, block averaging).

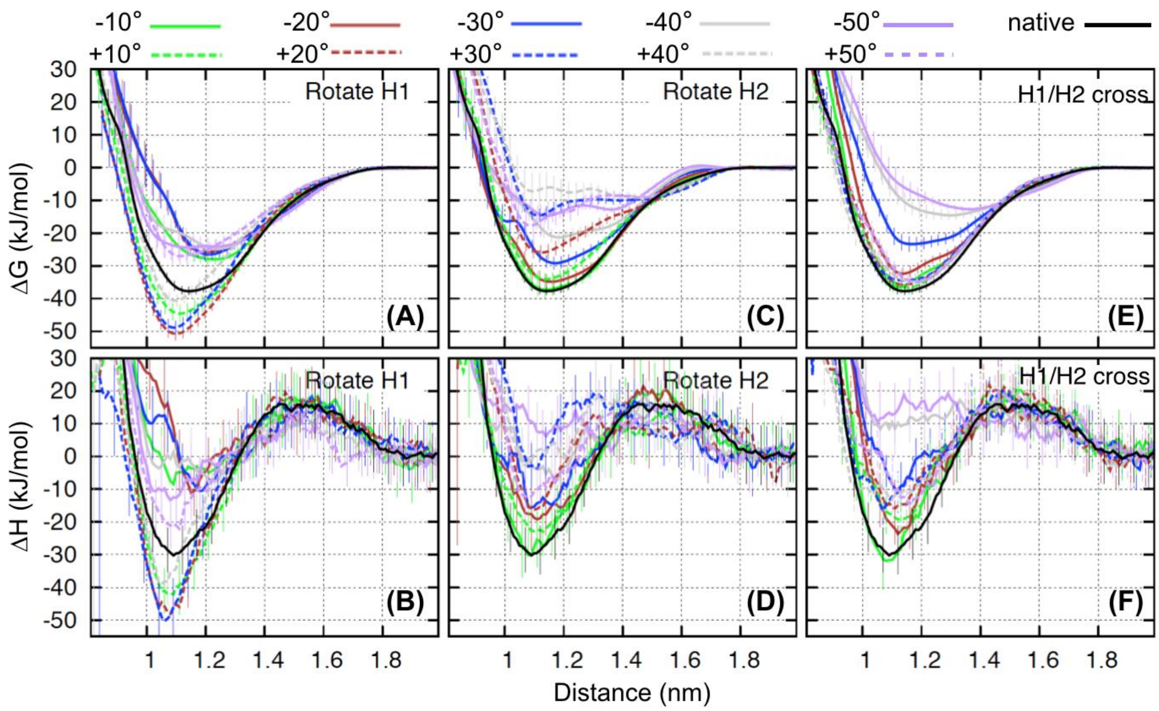

S2 Fig. Im9 energetic profiles for H1→H2. Inter-helical PMFs (A, C, E) and distance-dependent enthalpies (B, D, F) are shown for rotation of H1 (A, B), rotation of H2 (C, D), and changing of the H1-H2 crossing angle (E, F), while leaving the backbone native configuration of the opposing helix unchanged in the spatial coordinates of the simulation system. In each plot, data for native and nonnative packing angles are shown as black and colored curves, respectively. Colors for rotation or crossing angles are listed at the top of this figure, where negative and positive angular changes are indicated, respectively, by solid and dashed lines. Error bars show standard deviations of the mean estimated by block averaging.

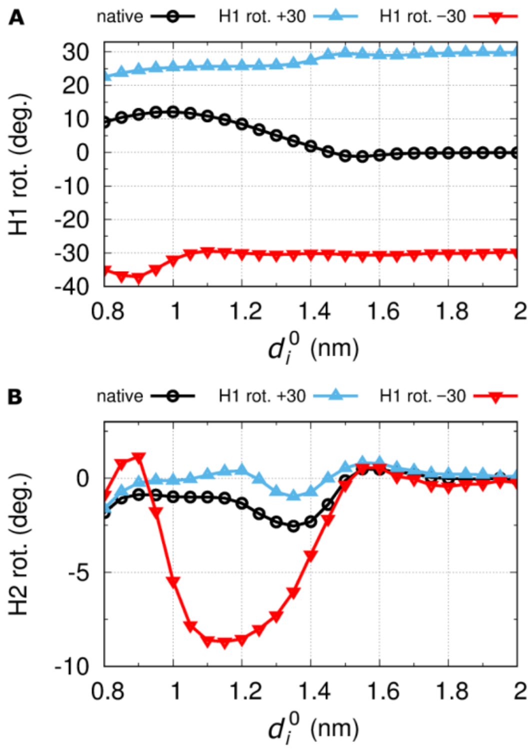

S3 Fig. Average helical rotation sampled during US-VREX simulation of the H1→H2 system. Data show *actual* rotation of (A) H1 and (B) H2 for native (black curve) and nonnative orientations with H1 rotation targeted to +30° (blue curve) or −30° (red curve). Deviations between actual and targeted rotations arise from effects of many potential energy terms in the simulated system in addition to the imposed angle-restraining potential. The differences between actual and target angles shown here are relative to baselines defined by the behavior of the system at $d_i^0 > 2.0$ nm for which the interactions between the two bundles are expected to be sufficiently weak such that they may be considered to be independent.

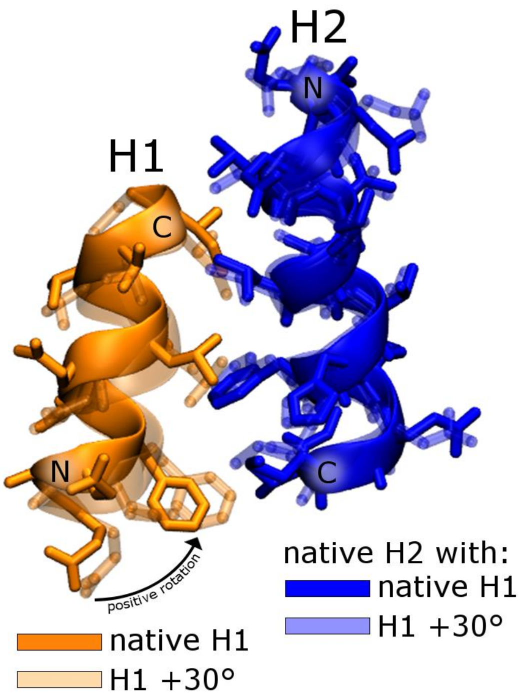

S4 Fig. Representative structures corresponding to free energy minima for the H1→H2 system. The structures are restrained to the native orientation (solid color) and a nonnative orientation with H1 rotated by +30° (translucent color). Free energy minima are located at helical separation distance *d* = 1.14 nm for the native orientation and *d* = 1.09 nm for the nonnative orientation with H1 rotated by +30°.

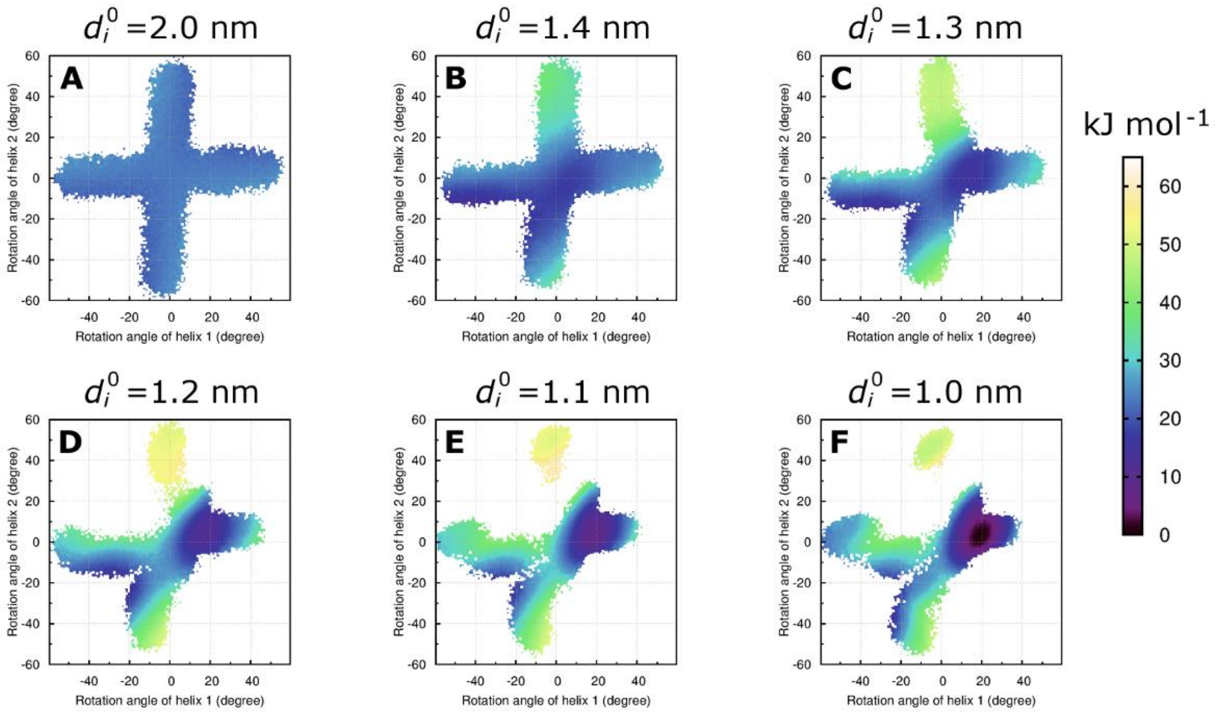

S5 Fig. Two-dimensional PMFs of the H1, H2 packing angles in H1→H2 simulations with helical rotation but no change in H1-H2 crossing angle. The format is similar to that of Fig. 2 in the main text. Data are for restrained inter-helical distances, d_i^0, from 2.0 nm to 1.0 nm as indicated above each plot. The color scale on the right is for the *relative* free energy at any given value of d_i^0, but the scale does not apply across different values of d_i^0. White regions have no sampling.

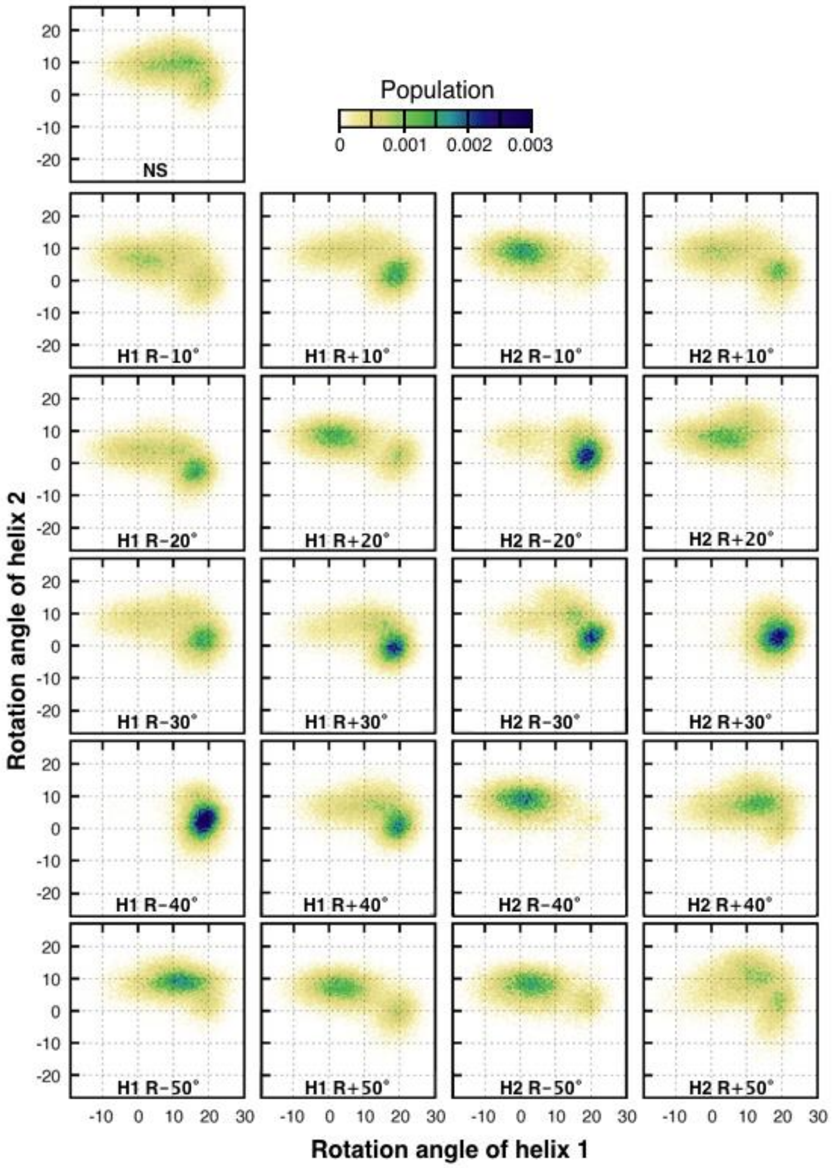

S6 Fig. Population density maps of Im9 H1 and H2 packing angles obtained from the single-chain H1^{L}H2 system. Each subplot represents an independent simulation that was initiated in either the native state (NS) or with the indicated helix rotated (R) by the specified angle.

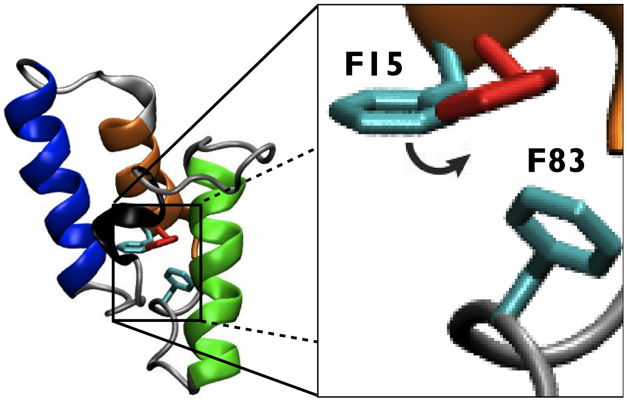

S7 Fig. A potential steric clash. Positive rotation of H1 brings Im9 H1 residue F15 into closer contact with C-terminal residue F83, leading to a likely steric clash if the C-terminal region retains its structure in the native state. Helices in the Im9 NMR structure (PDB ID: 1IMQ; see [2] of **S1 Text**) are colored as follows: H1, orange; H2, blue; H3, black; and H4, green; whereas intervening loops and C-terminus are in grey. Enlarged view (right): F15 and F83 side chains are shown as cyan sticks in the native configuration and the F15 side chain is shown in red after rotation of H1 by +30°.

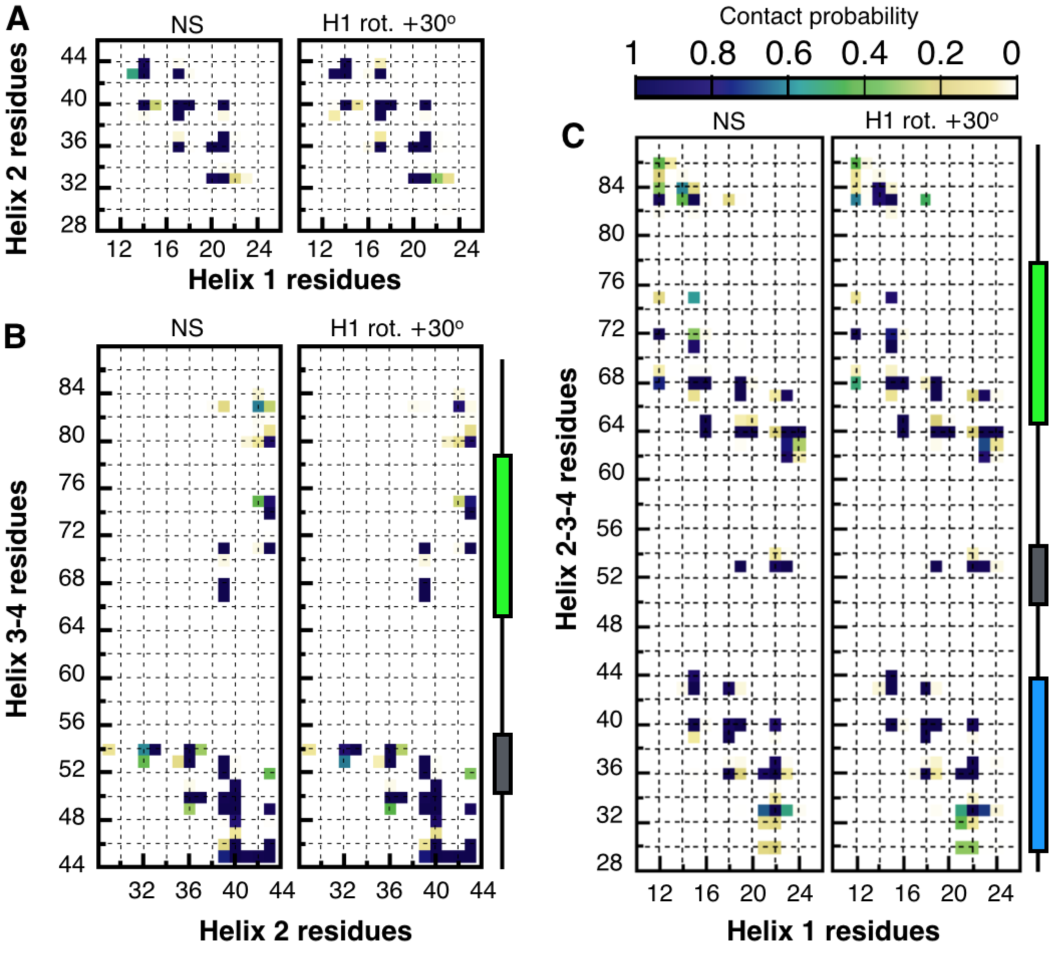

S8 Fig. Contact probability maps of Im9 helical association. (A) Contact probabilities for H1→H2 between residues in H1 and those in H2. Here a contact is said to exist between two residues if at least two heavy atoms, one from each residue, are separated by ≤ 0.45 nm. (B, C) Corresponding contact probabilities for H1→ $H2^L H3^L H4^C$ between residues in H2 and those in H3 and H4 (B), and between residues in H1 and those in H2, H3, and H4 (C). Color scale (top right) indicates a range from no contact (white for probability zero) to constant contact (blue for probability of one). In each of these cases (A, B, and C), results shown are for native (left panel) and nonnative rotation of H1 by +30° (right panel). For the H1→ $H2^L H3^L H4^C$ results in (B) and (C), residues of the helices are marked by color bars to the right of each set of contact maps (H2: blue, H3: grey, H4: green).

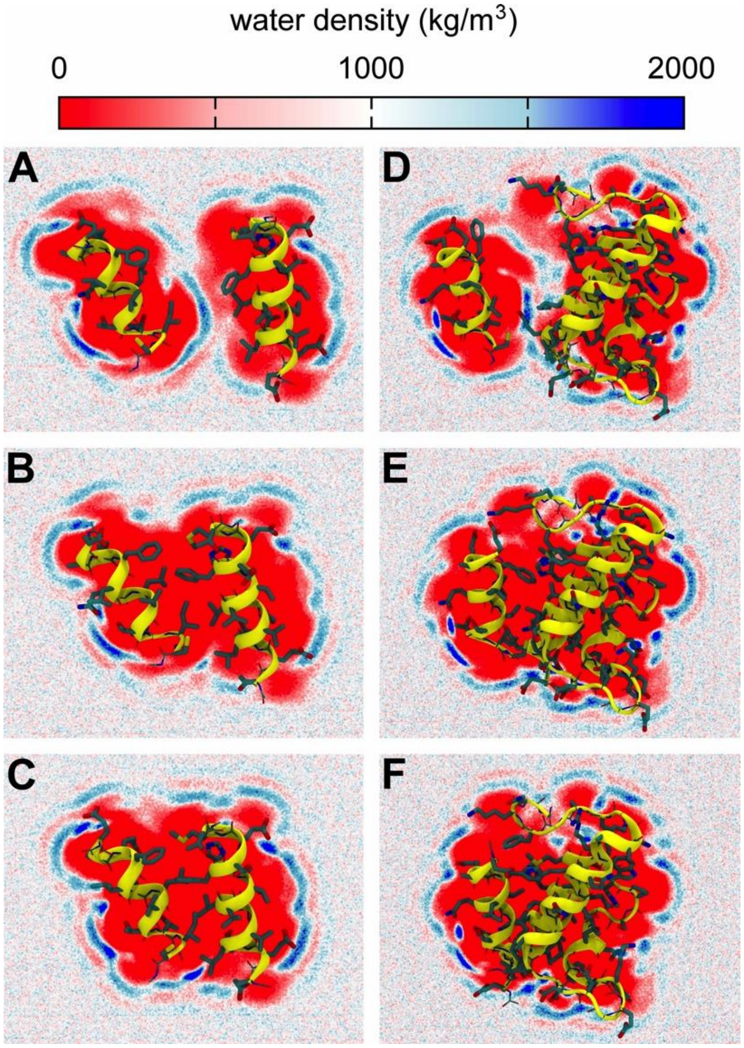

S9 Fig. Changes in local water density upon Im9 helix-helix binding. Colors (top scale) indicate densities that are greater (blue) or less (red) than bulk water at 300 K for a 0.4 nm slice passing through the center of mass of H1 and H2 (A, B, C) or $H2^{L}H3^{L}H4^{C}$ (D, E, F). Data shown depict three representative separations between the approaching helix bundles (cf. Fig. 6 of the main text): (A, D) the position corresponding to the solvent-separated enthalpy minimum at d = 1.90 nm, (B, E) the desolvation enthalpic barrier at d = 1.45 nm, and (C, F) the free energy minimum at d = 1.15 nm. Note that the sidechains of the approaching helix bundles are farther apart at the desolvation enthalpic barrier (B, E) than at contact (C, F). However, unlike the situation in (A, B), there is no water between the helix bundles in (B, E). Thus the total system volume is larger for (B, E) than for either (A, B) or (C, F). In other words, a volume barrier develops around d = 1.45 nm for both the H1→H2 and H1→$H2^{L}H3^{L}H4^{C}$ systems (see Fig. 6G, H of the main text).

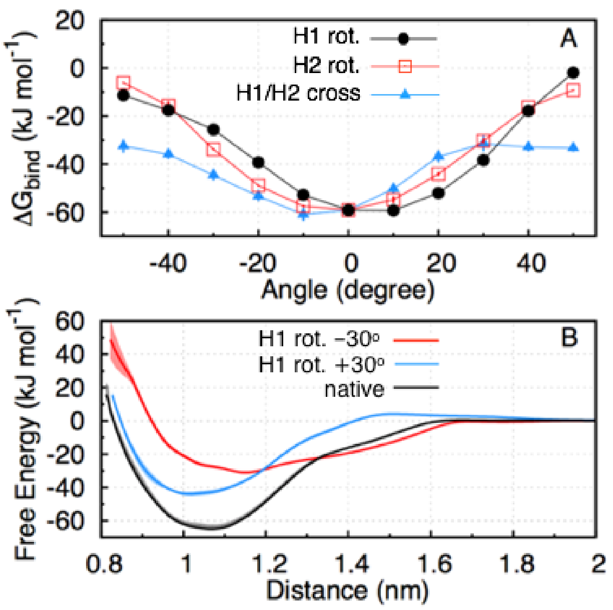

S10 Fig. Im7 binding free energies for H1→H2. (A) Binding free energies, ΔG_{bind}, for the association of H1 and H2 with native and nonnative packing angles. Nonnative configurations are generated by rotating H1 (filled black circles), or H2 (open red squares), or changing the H1-H2 crossing angle (filled blue triangles). ΔG_{bind} is computed by integrating the PMF over a free-energy basin as in Fig. 2A and Fig. 3A of the main text. (B) PMFs shown are distance-dependent free energies for the association of H1 and H2 in native (black curve) and nonnative orientations with H1 rotated by +30° (blue curve) or −30° (red curve). Standard deviations of the mean from block averaging are shown as vertical bars in (A) or shaded regions in (B). Im7 native state is from PDB 1AYI (ref. [1] of **S1 Text**), with H1 and H2 comprising residues 12-26 and 32-45, respectively, as determined by DSSP (ref. [20] of **S1 Text**).

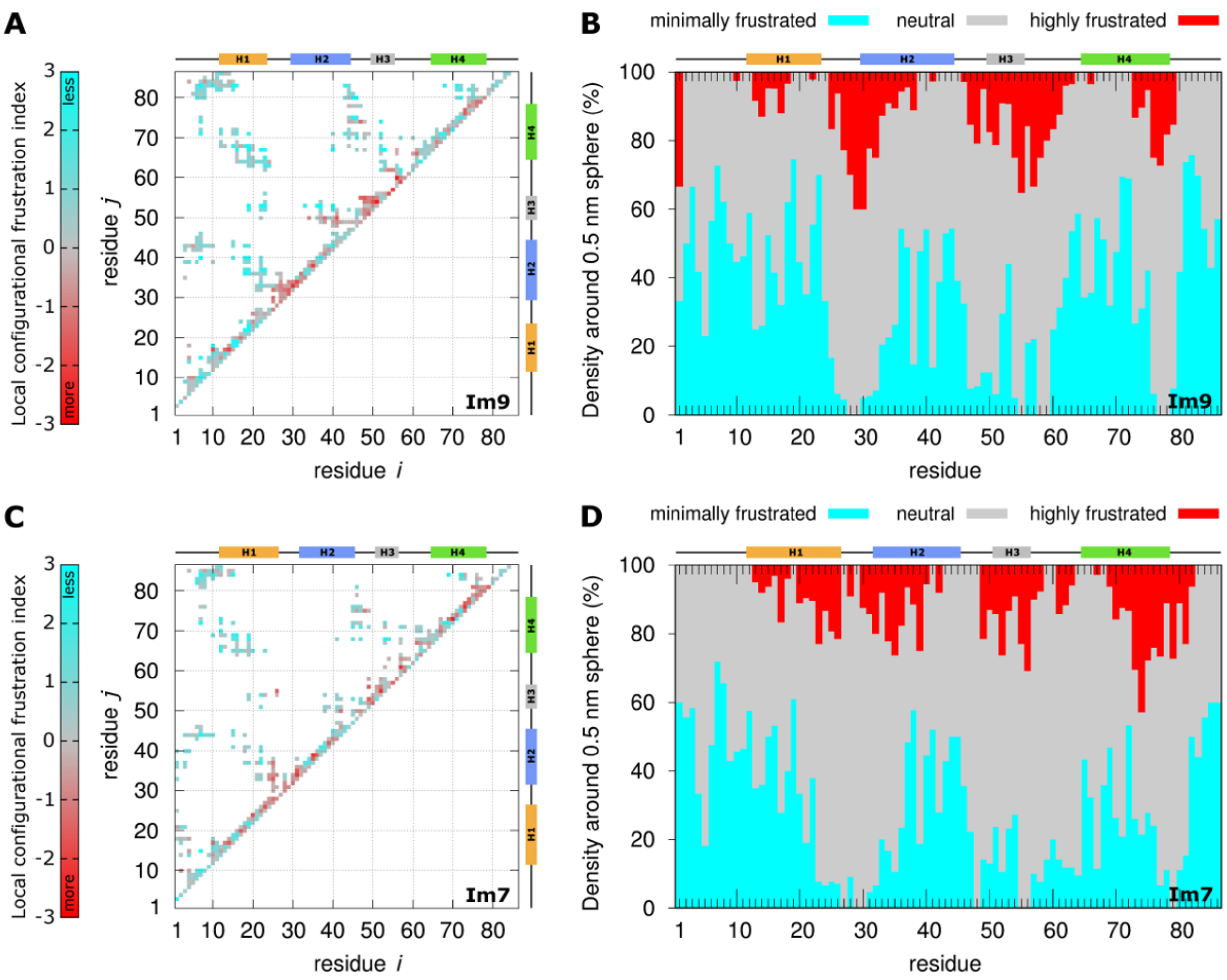

S11 Fig. Localized frustration computed by Protein Frustratometer 2. Data shown for (A, B) Im9 based on PDB 1IMQ (2) and (C, D) Im7 based on PDB 1AYI. (A, C) Configurational frustration index, F^c, for native state contacts. Frustration increases as F^c decreases. (B, D) Stacked histograms showing proportion of contacts within 0.5 nm that are minimally frustrated (cyan; $F^c > 0.78$), neutral (grey), or highly frustrated (red; $F^c < -1$). The positions of the four Im9/Im7 helices are shown in the same color code as in the other figures in this study. Data are computed by Protein Frustratometer 2 (ref. [19] of **S1 Text**) without electrostatics, the inclusion of which does not affect the results significantly. C-terminal Im9 residue Gly87 is omitted because it is not resolved in the 1AYI crystal structure.

SUPPORTING REFERENCES

1. Dennis CA, Videler H, Pauptit RA, Wallis R, James R, Moore GR, et al. A structural comparison of the colicin immunity proteins Im7 and Im9 gives new insights into the molecular determinants of immunity-protein specificity. Biochem J. 1998;333(1):183-91.
2. Osborne MJ, Breeze AL, Lian L-Y, Reilly A, James R, Kleanthous C, et al. Three-dimensional solution structure and 13C nuclear magnetic resonance assignments of the Colicin E9 immunity protein Im9. Biochemistry. 1996;35(29):9505-12. doi: 10.1021/bi960401k.
3. Ferrenberg AM, Swendsen RH. New Monte Carlo technique for studying phase transitions. Phys Rev Lett. 1988;61(23):2635-8.
4. Hansmann UHE. Parallel tempering algorithm for conformational studies of biological molecules. Chem Phys Lett. 1997;281(1-3):140-50. doi: 10.1016/s0009-2614(97)01198-6.
5. Sugita Y, Okamoto Y. Replica-exchange molecular dynamics method for protein folding. Chem Phys Lett. 1999;314(1-2):141-51. doi: 10.1016/s0009-2614(99)01123-9.
6. Tesi MC, Janse van Rensburg EJ, Orlandini E, Whittington SG. Monte carlo study of the interacting self-avoiding walk model in three dimensions. J Stat Phys. 1996;82(1):155-81. doi: 10.1007/bf02189229.
7. Hukushima K, Nemoto K. Exchange Monte Carlo method and application to spin glass simulations. J Phys Soc Jpn. 1996;65(6):1604-8.
8. Rauscher S, Neale C, Pomès R. Simulated tempering distributed replica sampling, virtual replica exchange, and other generalized-ensemble methods for conformational sampling. J Chem Theory Comput. 2009;5(10):2640-62. doi: 10.1021/ct900302n.
9. Neale C, Madill C, Rauscher S, Pomès R. Accelerating Convergence in Molecular Dynamics Simulations of Solutes in Lipid Membranes by Conducting a Random Walk along the Bilayer Normal. J Chem Theory Comput. 2013;9(8):3686-703.
10. Grossfield A. WHAM: the weighted histogram analysis method http://membrane.urmc.rochester.edu/content/wham (accessed Mar 21 2017).
11. Kumar S, Rosenberg JM, Bouzida D, Swendsen RH, Kollman PA. The weighted histogram analysis method for free-energy calculations on biomolecules. I. The method. J Comput Chem. 1992;13(8):1011-21. doi: 10.1002/jcc.540130812.
12. Flyvbjerg H, Petersen HG. Error estimates on averages of correlated data. J Chem Phys 1989;91(1):461-6.
13. Neale C, Bennett WFD, Tieleman DP, Pomès R. Statistical convergence of equilibrium properties in simulations of molecular solutes embedded in lipid bilayers. J Chem Theory Comput. 2011;7(12):4175-88.
14. Roux B. The calculation of the potential of mean force using computer simulations. Comput Phys Commun. 1995;91(1-3):275-82.
15. Souaille M, Roux B. Extension to the weighted histogram analysis method: combining umbrella sampling with free energy calculations. Comput Phys Commun. 2001;135(1):40-57.
16. Kumar S, Rosenberg JM, Bouzida D, Swendsen RH, Kollman PA. Multidimensional free-energy calculations using the weighted histogram analysis method. J Comput Chem. 1995;16(11):1339-50.

17. MacCallum JL, Moghaddam MS, Chan HS, Tieleman DP. Hydrophobic association of alpha-helices, steric dewetting and enthalpic barriers to protein folding. Proc Natl Acad Sci USA 2007;104(15):6206-10.
18. Eisenhaber F, Lijnzaad P, Argos P, Sander C, Scharf M. The double cubic lattice method: efficient approaches to numerical integration of surface area and volume and to dot surface contouring of molecular assemblies. J Comput Chem. 1995;16(3):273-84.
19. Parra RG, Schafer NP, Radusky LG, Tsai M-Y, Guzovsky AB, Wolynes PG, et al. Protein Frustratometer 2: A tool to localize energetic frustration in protein molecules, now with electrostatics. Nucl Acids Res. 2016;44(W1):W356-W60.
20. Kabsch W, Sander C. Dictionary of protein secondary structure: pattern recognition of hydrogen-bonded and geometrical features. Biopolymers. 1983;22(12):2577-637. Epub 1983/12/01. doi: 10.1002/bip.360221211. PubMed PMID: 6667333.
